# IL-22 receptor signaling in Paneth cells is critical for their maturation, microbiota colonization, Th17-related immune responses, and anti-*Salmonella* immunity

**DOI:** 10.1038/s41385-020-00348-5

**Published:** 2020-10-15

**Authors:** Stephen J. Gaudino, Michael Beaupre, Xun Lin, Preet Joshi, Sonika Rathi, Patrick A. McLaughlin, Cody Kempen, Neil Mehta, Onur Eskiocak, Brian Yueh, Richard S. Blumberg, Adrianus W. M. van der Velden, Kenneth R. Shroyer, Agnieszka B. Bialkowska, Semir Beyaz, Pawan Kumar

**Affiliations:** 1 Department of Microbiology and Immunology, Renaissance School of Medicine, Stony Brook University, Stony Brook, NY USA; 2 Cold Spring Harbor Laboratory, Cold Spring Harbor, NY 11724 USA; 3 Department of Gastroenterology, Brigham and Womenʼs Hospital, Boston, MA 02115 USA; 4 Department of Pathology, Renaissance School of Medicine, Stony Brook University, Stony Brook, NY USA; 5 Department of Medicine, Renaissance School of Medicine, Stony Brook University, Stony Brook, NY USA

## Abstract

Interleukin-22 (IL-22) signaling in the intestines is critical for promoting tissue-protective functions. However, since a diverse array of cell types (absorptive and secretory epithelium as well as stem cells) express IL-22Ra1, a receptor for IL-22, it has been difficult to determine what cell type(s) specifically respond to IL-22 to mediate intestinal mucosal host defense. Here, we report that IL-22 signaling in the small intestine is positively correlated with Paneth cell differentiation programs. Our *Il22Ra1*^*fl/fl*^*;Lgr5-EGFP-cre*^*ERT2*^-specific knockout mice and, independently, our lineage-tracing findings rule out the involvement of Lgr5^+^ intestinal stem cell (ISC)-dependent IL-22Ra1 signaling in regulating the lineage commitment of epithelial cells, including Paneth cells. Using novel Paneth cell-specific IL-22Ra1 knockout mice (*Il22Ra1*^*fl/fl*^*;Defa6-cre*), we show that IL-22 signaling in Paneth cells is required for small intestinal host defense. We show that Paneth cell maturation, antimicrobial effector function, expression of specific WNTs, and organoid morphogenesis are dependent on cell-intrinsic IL-22Ra1 signaling. Furthermore, IL-22 signaling in Paneth cells regulates the intestinal commensal bacteria and microbiota-dependent IL-17A immune responses. Finally, we show ISC and, independently, Paneth cell-specific IL-22Ra1 signaling are critical for providing immunity against *Salmonella enterica* serovar Typhimurium. Collectively, our findings illustrate a previously unknown role of IL-22 in Paneth cell-mediated small intestinal host defense.

## Introduction

A critical role for IL-22 signaling via IL-22Ra1-STAT3 has been established in modulating mucosal immunity, microbiota colonization, inflammation, and tissue repair.^1–6^ Currently, recombinant human IL-22 IgG2-Fc (IL-22.Fc) fusion protein is in FDA-approved phase IIa clinical trials for graft vs host disease (trial ID NCT0246651) and alcoholic hepatitis (NCT 02655510). Despite the myriad of gastrointestinal responses to IL-22 stimulation, there have been limited findings that elucidate whether these responses to injury or infection are consequences of direct stimulation to functionally distinct absorptive or secretory epithelial cells or if they are stem cell-derived.

The protective and inflammatory roles of IL-22 in the intestinal tissue have been reported.^1–10^ This has been implicated by increased susceptibility of germ-line *Il22*^*−/−*^, or receptor *Il22Ra1*^*−/−*^, knockout mice to murine colitis models including dextran sodium sulfate- and bacteria-induced colitis.^1,2,4–7^ Furthermore, cell proliferative and tumorigenic effects of IL-22 have been documented through the use of *Il22bp*^*−/−*^ mice that lack IL-22 binding protein, an antagonistic receptor for IL-22.^10,11^ Although global knockouts provide insight into how IL-22 responses impact gut defense, the precise mechanisms regarding how IL-22 exerts these effects remain poorly understood. Numerous intestinal epithelial cell types such as absorptive enterocytes, secretory cells (goblet and Paneth) and intestinal stem cells (ISCs) express the IL-22Ra1 monomer required for IL-22 effector function, but how IL-22 stimulates these cell types is not well characterized.^12^

Paneth cells are critical for small intestinal host defense and their dysregulation results in microbiota-dependent inflammatory responses.^13–15^ In addition, Crohn’s patients have Paneth cell abnormalities, reduced α-defensin expression, and elevated IL-22 levels in inflamed tissues.^14,16,17^ Recent studies show that IL-22 acts via ISCs or transit-amplifying cells to promote epithelial cell regeneration.^12,18,19^ However, a direct role of IL-22 (via Lgr5) in regulating secretory cell lineage commitment and Paneth cell-specific responses is not known. It is possible that IL-22 either acts directly on Paneth cells or via ISCs or secretory progenitor cells to regulate their differentiation and effector functions, but how IL-22 regulates Paneth cell function has not clearly been investigated due to a lack of lineage-specific tools. Paneth cell-derived EGF, TGFβ, WNT3, WNT6, and WNT9b regulate, in part, ISC maintenance.^20–22^ Thus, it is unknown whether IL-22Ra1 signaling in Paneth cells reciprocally regulates the small intestinal niche of ISCs and epithelial cell differentiation.

It has been shown that IL-22 promotes colonic *Salmonella enterica* serovar Typhimurium (*S*. Typhimurium) colonization and that the microbiota secondarily mediates this effect.^23^ In the small intestine, however, IL-22 confers a protective effect against *Salmonella*.^11,24,25^ The importance of Paneth cells in controlling *S*. Typhimurium infection is evident by a study in human defensin 5 (HD5)-transgenic mice.^26^ While IL-22 may act via α-defensins to regulate *S*. Typhimurium colonization, the Paneth cell-specific role of IL-22 in regulating *S*. Typhimurium has not been investigated. Our study helps unravel these gaps in knowledge by providing evidence that IL-22Ra1 signaling in Paneth cells, but not ISCs, is required for their maturation, antimicrobial functions, microbiota colonization, and selective WNT secretion. Our data also suggest that both ISC- and, independently, Paneth cell-specific IL-22Ra1 signaling are required for providing immunity against *S*. Typhimurium.

## Results

### Paneth cell differentiation and their antimicrobial effector functions are positively correlated with IL-22 responses in the small intestine

**Fig. 1:**
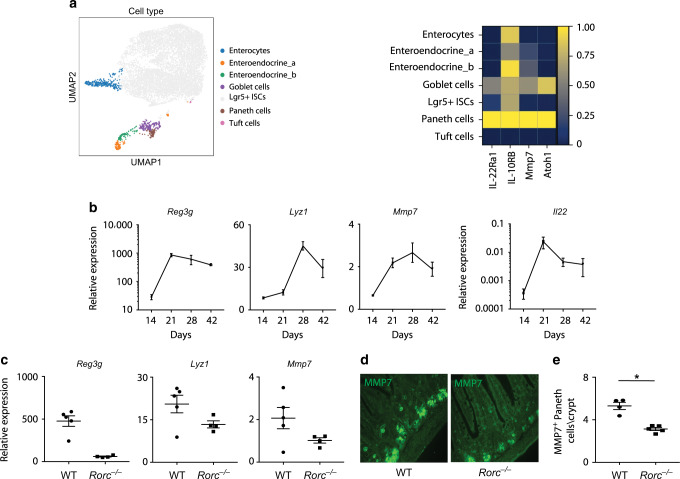
Paneth cell differentiation and effector functions are positively correlated with IL-22 responses in the small intestine. **a** Principal component analysis (left panel) and heatmap diagram (right panel) of single-cell primary small intestinal organoid RNA sequencing depicting the expression patterns of *Il22Ra1 *in epithelial and stem cell lineages. **b** Terminal ileum RT-PCR analysis displaying alterations in Paneth cell-specific genes as well as *Il22* expression over a period of 42 days post birth in naive C57BL/6 mice. **c** RT-PCR analysis of *Reg3γ*, *Lyz1* and *Mmp7* expression from ileal tissues of naive WT and *Rorc*^*−/−*^ mice. **d** Immunofluorescence staining for MMP7 from ilea tissue of naive WT and *Rorc*^*−/−*^ mice. **e** Number of MMP7^+^ cells from Fig. 1d. Figure 1c is representative of two independent experiments. Figure 1b is representative of at least 3–6 mice per group and was generated from two independent experiments. *P ≤ 0.05 (Mann–Whitney test, two-tailed).

Paneth cells play an important role in small intestinal host defense by regulating microbiota colonization and providing protection against pathogens.^14,26^ Likewise, IL-22 is a key player in mucosal host defense.^1–6^ All intestinal epithelial lineages including Paneth cells and ISCs express the IL-22 receptor;^19^ however, it is not known whether IL-22-dependent regulation of small intestinal host defense is dependent on ISCs or a specific epithelial lineage. To examine differences in gene expression among small intestinal enterocytes and secretory cells, single-cell RNA sequencing (scRNA-seq) was performed on naive C57BL/6 mouse-derived primary small intestinal organoids. Our scRNA-seq results demonstrated that Paneth cells express the highest level of *Il22ra1* when compared to ISCs as well as enterocyte, goblet, tuft, and endocrine lineages (Fig. 1a). We also found that expression patterns of key Paneth cell-specific genes, particularly *Mmp7* and *Lyz1*, were positively correlated with *Il22* expression in the terminal ileum of naive C57BL/6 mice at different days after birth (Fig. 1b). These data suggest that IL-22 may influence Paneth cell differentiation, maintenance, and antimicrobial activities. Using naive *Rorc*^*−/−*^ mice which lack IL-22 and several other cytokines including IL-17A, we found that *Lyz1, Mmp7*, and *Reg3γ* (positive control) expression as well as Paneth cell number were substantially reduced in the terminal ileum of these mice (Fig. 1c–e). In line with our *Rorc*^*−/−*^ mice observation, we found a significant reduction of *Lyz1* and *Mmp7* expression in the terminal ileum of *Il22*^*−/−*^ mice compared to cohoused WT mice. Interestingly, Paneth cell number was modestly reduced, albeit not significant, in *Il22*^*−/−*^ mice (Supplementary Fig. 1a, b). Collectively, these data suggest that IL-22 influences Paneth cell antimicrobial effector function by regulating their expression of key antimicrobial peptides.

### Intestinal IL-22Ra1 deficient mice have Paneth cell defects

**Fig. 2:**
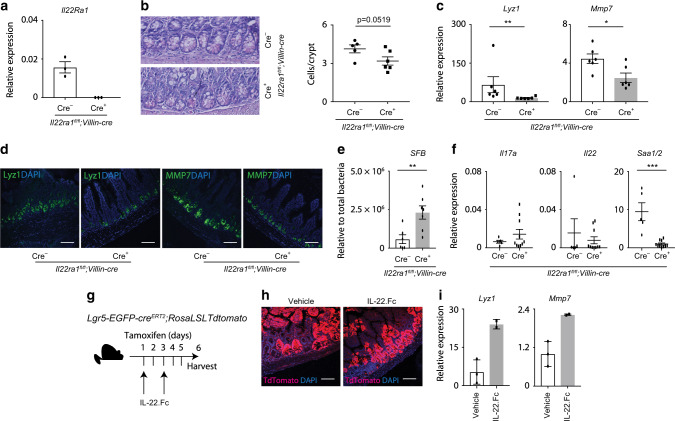
Intestinal epithelium-specific IL-22Ra1 knockout mice display Paneth cell defects. **a** RT-PCR data depicting *Il22Ra1* expression in the terminal ileum of naive *Il22Ra1*^*fl/fl*^*;Villin-cre*+*/−* mice. **b** Representative phloxine-tartrazine staining image (left panel) and average Paneth cell number/crypt (right panel) in the ileum of naive *Il22Ra1*^*fl/fl*^*;Villin-cre*+*/−* mice. **c** RT-PCR analysis of *Lyz1* and *Mmp7* expression from ileal tissues of naive *Il22Ra1*^*fl/fl*^*;Villin-cre*+*/−* mice. **d** Representative immunofluorescence image of Lyz1 and MMP7 in the ileal tissue of naive *Il22Ra1*^*fl/fl*^*;Villin-cre*+*/−* mice. **e** RT-PCR analysis of SFB levels in the terminal ileum of naive *Il22Ra1*^*fl/fl*^*;Villin-cre*+*/−* mice. **f** RT-PCR analysis of *Il17a*, *Il22* and *Saa1/2* expression from the terminal ileum of naive *Il22Ra1*^*fl/fl*^*;Villin-cre*+*/−* mice. **g** Layout of *Lgr5-EGFP-cre*^*ERT2*^*;RosaLSLtdTomato* treatment with tamoxifen and either vehicle (PBS) or IL-22.Fc (80 μg/mouse). **h** Representative immunofluorescence images of TdTomato expression in vehicle or IL-22.Fc treated *Lgr5-EGFP-cre*^*ERT2*^*;RosaLSLtdTomato* mice. **i** RT-PCR analysis of *Lyz1* and *Mmp7* expression in the terminal ileum of these mice. Figure 2b–f were generated from two independent experiments. Figure 2d is representative of at least four mice in each group Figure 2h, i are generated form 2–3 mice per group. Data are presented as mean ± SEM on all graphs except Fig. 2i (mean ± SD). Scale bars in relevant figures equal 100 μm. *P ≤ 0.05; **P ≤ 0.01; ***P ≤ 0.001 (Mann–Whitney test, two-tailed).

To further validate a role for IL-22 in regulating Paneth cell differentiation and antimicrobial activity, we utilized and validated (via RT-PCR and flow cytometry) intestinal epithelium-specific IL-22Ra1 knockout mice (Fig. 2a and Supplementary Fig. 1c). Terminal ilea of naive *Il22Ra1*^*fl/fl*^*;Villin-cre*+ and littermate cre- control mice were analyzed for Paneth cell numbers and expression levels of *Lyz1* and *Mmp7*. Our data show that Paneth cell number was modestly decreased in *Il22Ra1*^*fl/fl*^*;Villin-cre*+ mice (Fig. 2b). Interestingly, *Lyz1* and *Mmp7* expression (RNA and protein) were significantly reduced in the terminal ileum of *Il22Ra1*^*fl/fl*^*;Villin-cre*+ mice (Fig. 2c, d). It has been reported that *Il22*^*−/−*^ and *Mmp7*^*−/−*^ mice possess a commensal dysbiosis indicated by an increased colonization of segmented filamentous bacteria (SFB) in the small intestine.^3,27^ As expected, we detected increased colonization of SFB in terminal ileum luminal contents of *Il22Ra1*^*fl/fl*^*;Villin-cre*+ mice (Fig. 2e). SFB colonization regulates small intestinal IL-17A and IL-22 responses in a serum amyloid A (SAA)-dependent manner.^28^ Despite increased levels of SFB colonization in *Il22Ra1*^*fl/fl*^*;Villin-cre*+ mice, we did not find any difference in *Il17a* and *Il22* transcript levels in the terminal ileum (Fig. 2f). This could be related to a reduced expression of *Saa1/2* in the terminal ileum of *Il22Ra1*^*fl/fl*^*;Villin-cre*+ mice (Fig. 2f). Our data suggest that IL-22Ra1 signaling in the small intestine plays an important role in regulating Paneth cell antimicrobial gene expression, which could promote increased SFB colonization. However, it remains unclear whether defects in Paneth cell function are due to a reduced number of Paneth cells (compromised ISC function) or a functional impact of IL-22Ra1 signaling on Paneth cells. To test the effects of IL-22 on ISC-mediated lineage commitment, we utilized *Lgr5-EGFP-cre*^*ERT2*^*;RosaLSLtdTomato* lineage tracer mice. We administered IL-22.Fc (half-life 48 h) as depicted in Fig. 2g and harvested small intestinal tissues 1 day after the last tamoxifen injection. Our data show no difference in tdTomato stained cells in the control or treatment groups suggesting IL-22 may not influence ISC-mediated intestinal epithelial cell lineage commitment (Fig. 2h). Interestingly, *Lyz1* and *Mmp7* expression was increased in the IL-22.Fc administered group (Fig. 2i). We additionally examined whether IL-22 acts on ISCs and Paneth cells. Using the described gating strategy (Supplementary Fig. 1d), we show that both ISCs and, possibly, Paneth cells respond to IL-22 stimulation as revealed by sustained intracellular pSTAT3 staining (Supplementary Fig. 1e), although additional work with an acute stimulation of IL-22 may be required. It is possible that IL-22 may act on Paneth cells to modulate their effector gene expression. The gain of function with the administration of IL-22.Fc has an inherent caveat since it does not mimic the physiological concentration of IL-22 in a defined crypt environment. Therefore, exacerbated IL-22 activation on non-ISC epithelial cell types may have an impact on stem and progenitor cells. To ideally study the effects of IL-22 on stem cells under homeostatic conditions, we have additionally utilized ISC-specific IL-22Ra1 knockout mice.

### IL-22Ra1 signaling in Lgr5^+^ ISCs is dispensable for the development of secretory cells including Paneth cells and antimicrobial activity

**Fig. 3:**
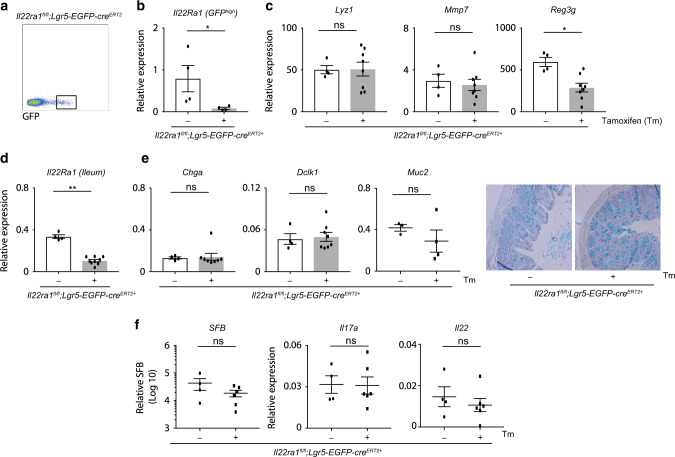
IL-22Ra1 signaling in intestinal stem cells is dispensable for lineage commitment and effector functions of secretory cells including Paneth cells. **a** Flow cytometry gating strategy utilized for sorting ISCs (GFP^high^) from the small intestine of naive *Il22Ra1*^*fl/fl*^*;Lgr5-EGFP-cre*^*ERT2+*^ mice. **b** RT-PCR analysis of *Il22Ra1* expression from sorted GFP^high^ cells. **c** RT-PCR analysis of *Lyz1*, *Mmp7* and *Reg3γ* expression from ileal tissue of naive *Il22Ra1*^*fl/fl*^*;Lgr5-EGFP-cre*^*ERT2+*^ mice treated with or without tamoxifen. **d** RT-PCR analysis of *Il22Ra1* expression in the ileal tissue of naive *Il22Ra1*^*fl/fl*^*;Lgr5-EGFP-cre*^*ERT2*^ mice treated with or without tamoxifen. **e** RT-PCR analysis of *Chga*, *Dclk1* and *Muc2* expression (left panel) and representative Alcian blue staining (right panel) from ileal tissues of naive *Il22Ra1*^*fl/fl*^*;Lgr5-EGFP-cre*^*ERT2+*^ mice treated with or without tamoxifen. **f** RT-PCR analysis of SFB levels as well as *Il17a* and *Il22* expression in the terminal ileum of naive *Il22Ra1*^*fl/fl*^*;Lgr5-EGFP-cre*^*ERT2+*^ mice treated with or without tamoxifen. Figure 3a is representative of two independent experiments. Figure 3b–f were generated from two independent experiments. Figure 3e right panel figure is representative of at least four mice in each group. Data are presented as mean ± SEM on relevant graphs. *P ≤ 0.05; **P ≤ 0.01 (Mann–Whitney test, two-tailed).

To test the effect of IL-22 on ISCs, we generated tamoxifen-inducible Lgr5^+^ ISC-specific Il22Ra1 mice by crossing *Il22Ra1*^*fl/fl*^ *and Lgr5-EGFP-cre*^*ERT2*^ mice. IL-22Ra1 deletion in Lgr5^+^ ISCs was confirmed by RT-PCR analysis of sorted GFP^high^ (Lgr5^+^) cells as well as IL-22Ra1 protein staining in tamoxifen-administered mice (Fig. 3a, b and Supplementary Fig. 2a). In contrast to our observation in *Il22Ra1*^*fl/fl*^*;Villin-cre*+ mice, we found no difference in Paneth cell number and *Lyz1* and *Mmp7* expression in tamoxifen-administered naive *Il22Ra1*^*fl/fl*^*;Lgr5-EGFP-cre*^*ERT2+*^ mice compared to the control (corn oil) group (Fig. 3c, and Supplementary Fig. 2b, c). Interestingly, tamoxifen-administered mice displayed decreased expression of *Reg3γ* (Fig. 3c). Paneth cells possess a half-life >30 days; thus, it is possible that IL-22Ra1 expression in Paneth cells is not compromised in tamoxifen-administered *Il22Ra1*^*fl/fl*^*;Lgr5-EGFP-cre*^*ERT2+*^ mice. Tamoxifen-administered *Il22Ra1*^*fl/fl*^*;Lgr5-EGFP-cre*^*ERT2+*^ mice lost IL-22Ra1 in all descendant cells in a time-dependent manner. We found reduced *Il22Ra1* expression (50%) in the terminal ileum of *Il22Ra1*^*fl/fl*^*;Lgr5-EGFP-cre*^*ERT2+*^ mice on day 10 after the last tamoxifen injection (Fig. 3d). Based on published work, we expect that longer time periods will result in 100% deletion of IL-22Ra1 in all Lgr5 descendant cells including Paneth cells. These cells would then act the same as those from *Il22Ra1*^*fl/fl*^*;Villin-cre*+ mice (Fig. 2a); therefore, longer post-injection periods may not be useful for our model. However, analysis of other secretory lineages (goblet, endocrine and tuft cells) with fast turn-over rates (3–4 days) and transcription factors required for secretory (ATOH1) or absorptive (HES1) epithelial cell lineage commitment in *Il22Ra1*^*fl/fl*^*;Lgr5-EGFP-cre*^*ERT2+*^ mice is informative. We found no difference in *Hes1* and *Atoh1* expression in sorted Lgr5^+^ cells of naive *Il22Ra1*^*fl/fl*^*;Lgr5-EGFP-cre*^*ERT2+*^ mice (Supplementary Fig. 2d). In addition, expression patterns of goblet cell (*Muc2*), endocrine cell (*Chga*), and tuft cell (*Dclk1*)-specific genes, as well as Alcian blue+ goblet cell numbers, were similar in tamoxifen-administered naive *Il22Ra1*^*fl/fl*^*;Lgr5-EGFP-cre*^*ERT2+*^ mice when compared to their control group (Fig. 3e). Comparable SFB colonization levels as well as *Il17a* and *Il22* responses were observed in tamoxifen administered to naive *Il22Ra1*^*fl/fl*^*;Lgr5-EGFP-cre*^*ERT2+*^ mice when compared to their control group (Fig. 3f). These results collectively indicate that IL-22 responses in Lgr5^+^ ISCs are dispensable for intestinal secretory cell lineage commitment under homeostatic conditions. Furthermore, these results are consistent with the possibility that IL-22 has an impact on Paneth cells to mediate small intestinal mucosal host defense.

### Intrinsic IL-22Ra1 signaling of Paneth cells is required for their effector functions

**Fig. 4:**
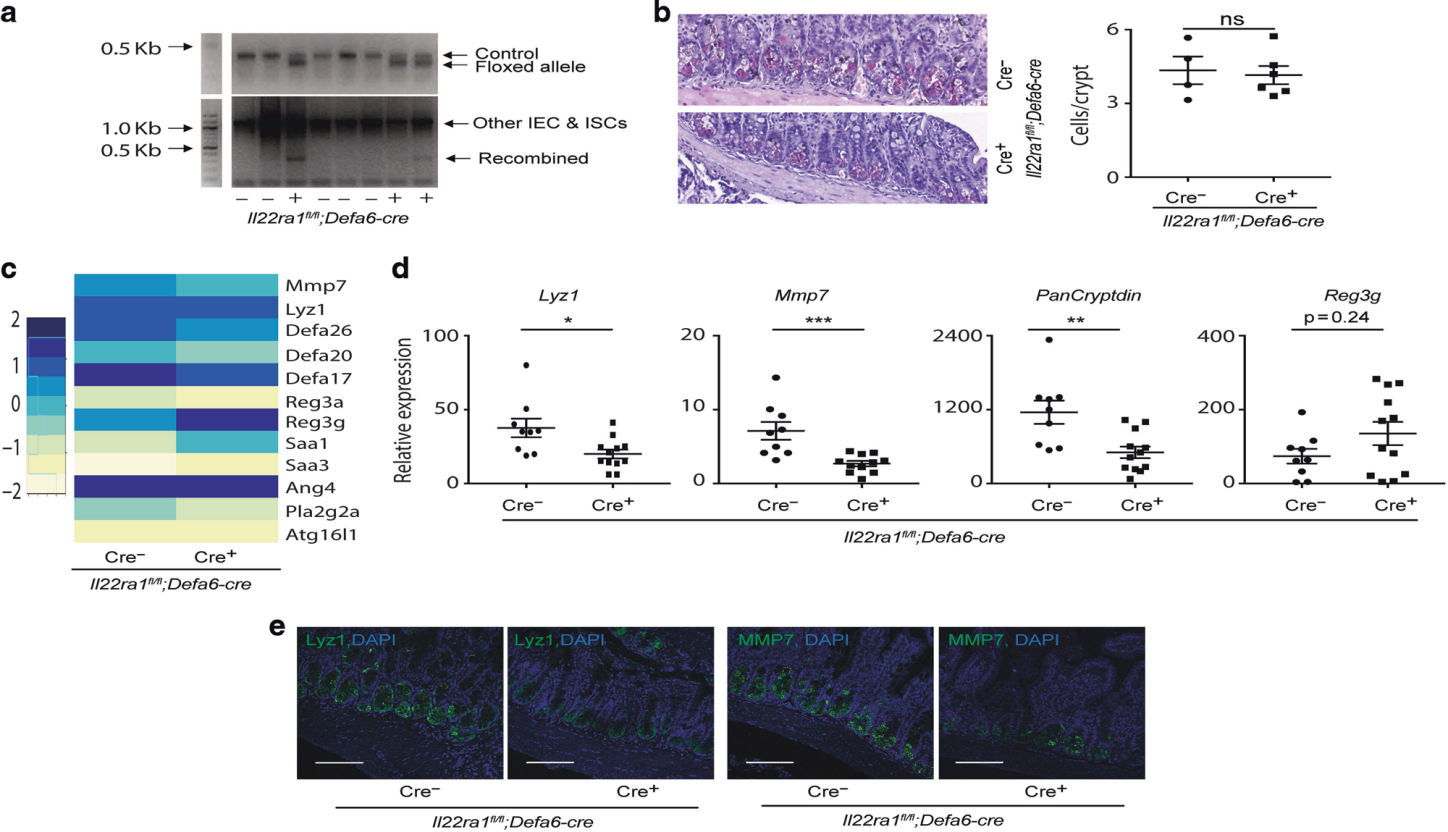
Paneth-specific IL-22Ra1 knockout mice display defects in Paneth cells. **a** Agarose gel PCR showing genotype and recombination of IL-22Ra1 allele in the target tissue of naive *Il22Ra1*^*fl/fl*^*;Defa6-cre*+*/−* mice. **b** Representative phloxine-tartrazine staining image (left panel) and average Paneth cell number/crypt (right panel) in the ileum of naive *Il22Ra1*^*fl/fl*^*;Defa6-cre*+*/−* mice. **c** RNA sequencing heat-map data showing the expression of selective genes in the terminal ileum of naive *Il22Ra1*^*fl/fl*^*;Defa6-cre*+*/−* mice. **d** RT-PCR analysis of *Lyz1, Mmp7, Pancryptdin* and *Reg3γ* expression from terminal ileal tissues of naive *Il22Ra1*^*fl/fl*^*;Defa6-cre*+*/−* mice. **e** Immunofluorescence analysis of Lyz1 and MMP7 from ileal tissue of naive *Il22Ra1*^*fl/fl*^*;Defa6-cre*+*/−* mice. Figure 4a is representative of two independent experiments. Figure 4b, d, e are generated from three independent experiments. Figure 4c is representative of 3 mice in each group. Figure 4e is representative of at least four mice in each group. Data are presented as mean ± SEM on relevant graphs. Scale bars in relevant figures equal 100 μm. *P ≤ 0.05; **P ≤ 0.01; ***P ≤ 0.001 (Mann–Whitney test, two-tailed).

Our findings in *Il22Ra1*^*fl/fl*^*;Villin-cre* and *Il22Ra1*^*fl/fl*^*;Lgr5-EGFP-cre*^*ERT2*^ mice suggest an impact of IL-22Ra1 signaling on Paneth cell for regulating their maintenance and antimicrobial activity. To test this, we generated Paneth cell-specific IL-22Ra1 knockout mice (*Il22Ra1*^*fl/fl*^*;Defa6-cre*) by crossing *Il22Ra1*^*fl/fl*^ and *Defa6-cre*+ mice. Knockdown of IL-22Ra1 on target cells was confirmed via PCR by using recombination-specific primers and via flow cytometry by using an IL-22Ra1-specific antibody (Fig. 4a and Supplementary Fig. 3). Interestingly, phloxine-tartrazine staining revealed no difference in the number of Paneth cells in the terminal ileum of naive *Il22Ra1*^*fl/fl*^*;Defa6-cre*+ mice (Fig. 4b). Next, we performed RNA sequencing (RNA-seq) of the terminal ileum of naive *Il22Ra1*^*fl/fl*^*;Defa6-cre*+ and littermate cre- mice. Sequencing data revealed reduced expression of Paneth cell-specific *Lyz1, Mmp7*, and select *α-defensin* antimicrobial peptides but increased expression of IL-22 inducible *Reg3γ* and serum amyloid genes (*Saa1* and *Saa3*) in the terminal ileum of naive *Il22Ra1*^*fl/fl*^*;Defa6-cre*+ mice (Fig. 4c). We confirmed RNA-seq results for the reduced expression for *Lyz1, Mmp7*, and *PanCryptdin* by RT-PCR (Fig. 4d). Reduced *Lyz1* and *Mmp7* expression was confirmed at the protein level using specific antibodies (Fig. 4e). Our data suggest that IL-22Ra1 signaling in Paneth cells is dispensable for their development and differentiation but required for their maturation and expression of antimicrobial effector genes.

### IL-22Ra1 signaling in Paneth cells is required for the expression of select WNTs and organoid growth but is dispensable for the maintenance of ISCs niche

**Fig. 5:**
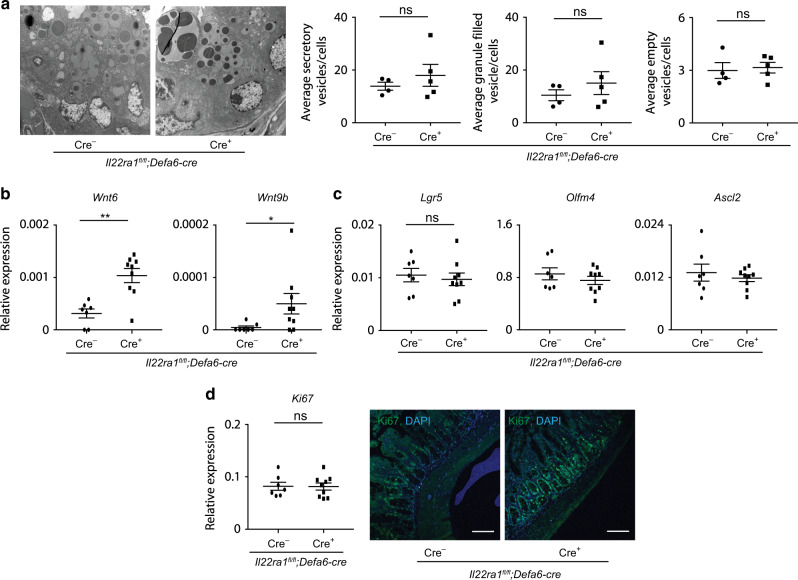
Dysregulated expression of WNTs are present in Paneth cell-specific IL-22Ra1 knockout mice. **a** Representative TEM images of Paneth cells (left panel) and number of secretory vesicles (right panel) in the terminal ileum of naive *Il22Ra1*^*fl/fl*^*;Defa6-cre*+*/−* mice. **b** RT-PCR analysis of *Wnt6* and *Wnt9b* expression from terminal ileal tissues of naive *Il22Ra1*^*fl/fl*^*;Defa6-cre*+*/−* mice. **c** RT-PCR analysis of *Lgr5, Olfm4* and *Ascl2* expression from terminal ileal tissues of naive *Il22Ra1*^*fl/fl*^*;Defa6-cre*+*/−* mice. **d** RT-PCR and immunofluorescence analysis of *Ki67* expression (left panel) and number of proliferative cells (right panel) from terminal ileal tissues of naive *Il22Ra1*^*fl/fl*^*;Defa6-cre*+*/−* mice. Figure 5b, c, d are generated from two independent experiments. Figure 5a is representative of at least 4–5 mice in each group. Data are presented as mean ± SEM on relevant graphs. Scale bars in relevant figures equal 100 μm. *P ≤ 0.05; **P ≤ 0.01 (Mann–Whitney test, two-tailed).

**Fig. 6:**
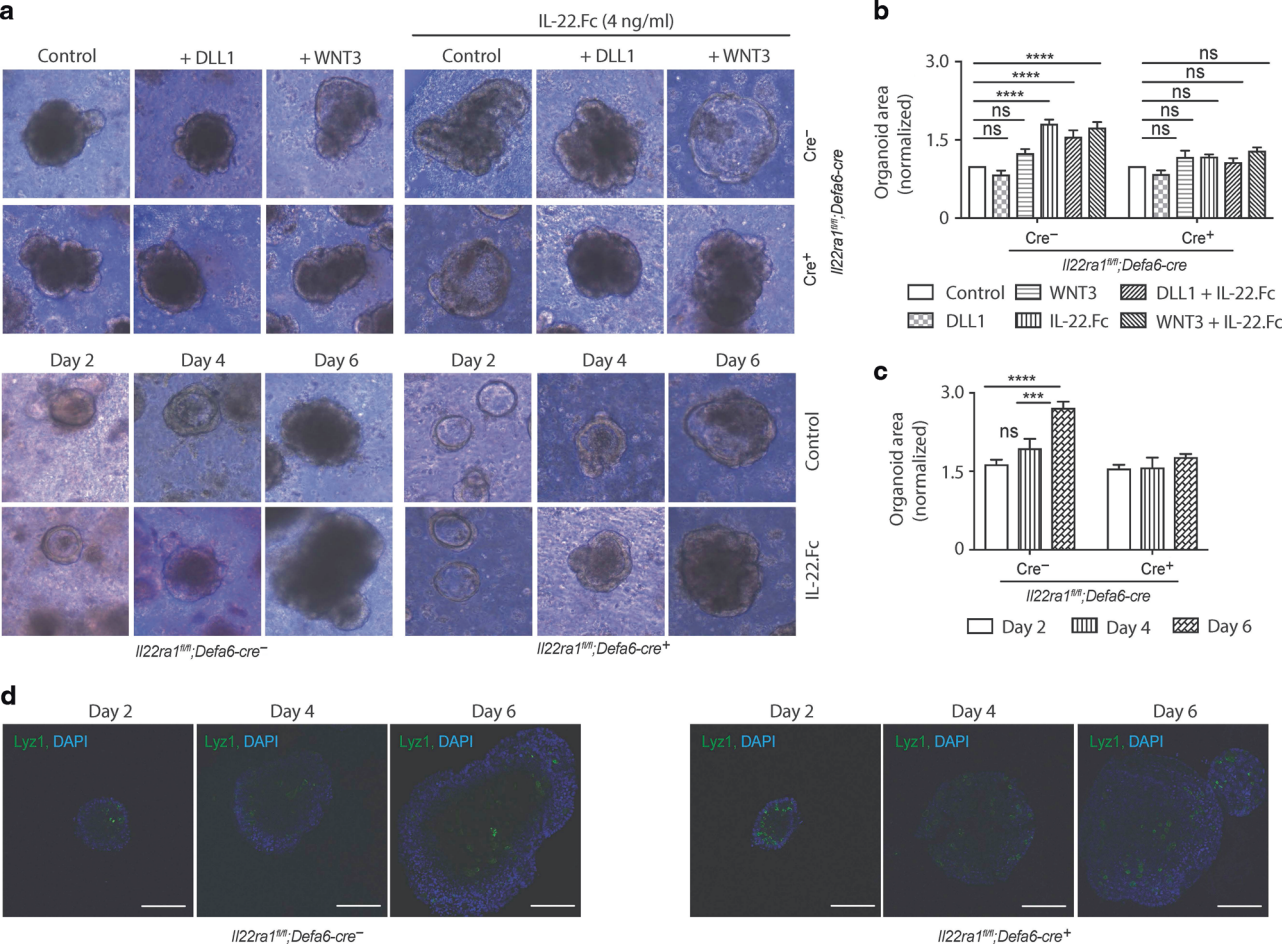
IL-22-mediated organoid growth is regulated by Paneth cell-specific IL-22Ra1 signaling. **a** Representative day 6 images of naive small intestinal *Il22Ra1*^*fl/fl*^*;Defa6-cre*+*/−* organoids stimulated with either 0 or 4 ng/mL IL-22.Fc in the presence or absence of 100 ng/mL Wnt3a or 1 μg/mL Dll1 over a 6-day period. **b** Normalized fold change of the area of organoids after 6 days of stimulation with either 0 ng/mL or 4 ng/mL IL-22.Fc in the presence or absence of 100 ng/mL Wnt3a or 1 μg/mL Dll1. **c** Normalized fold change of the area of *Il22Ra1*^*fl/fl*^*;Defa6-cre*+*/−* organoids after 2, 4 and 6 days of stimulation with either 0 or 4 ng/mL IL-22.Fc. **d** Representative confocal images of Lyz stained *Il22Ra1*^*fl/fl*^*;Defa6-cre*+*/−* organoids after 2, 4 and 6 days of growth. Organoid area was measured in ImageJ. The average area size of respective treated organoids was used to calculate the fold changes over the control group. Figure 6a–c are generated from two independent experiments. Figure 6a is representative of 4–5 mice in each group. Figure 6b is representative of 3–5 mice. Representative images in Fig. 6d are based on two mice in each group. Data are presented as mean ± SEM on relevant graphs. Scale bars in relevant figures equal 100 μm. ***P ≤ 0.001; ****P ≤ 0.0001 (two-way ANOVA).

Expression patterns of many Paneth cell proteins including α-defensins are considered constitutive and depend on cellular differentiation programs within the Paneth cells.^27^ Having found reduced Paneth cell effector proteins but not Paneth cell number in naive *Il22Ra1*^*fl/fl*^*;Defa6-cre* mice, we predicted altered Paneth cell morphology in our knockout mice. We performed TEM analysis in the terminal ileum of naive *Il22Ra1*^*fl/fl*^*;Defa6-cre*+ and their littermate cre- mice. Our data revealed no difference in Paneth cell morphology and secretory vesicle number in Paneth cell-specific IL-22Ra1 knockout mice (Fig. 5a). Interestingly, we found a modest increase in the number of granule-filled vesicles of *Il22Ra1*^*fl/fl*^*;Defa6-cre*+ mice (Fig. 5a and Supplementary Fig. 4). Canonical WNT signaling is required for Paneth cell maturation and maintenance of ISCs niche. Paneth cells secrete several WNTs (WNT3, WNT6, and WNT9b) and other growth factors (EGF and TGFβ) which have been shown to regulate, at least in part, ISC maintenance *in vitro*.^20–22^ We have found increased expression of *Wnt6* and *Wnt9b* in the ileum of naive *Il22Ra1*^*fl/fl*^*;Defa6-cre*+ mice, suggesting IL-22 negatively regulates WNT secretion (Fig. 5b). Of note, *Wnt3a* expression was below the detection limit, and expression patterns of *Tgfb1* and *Egf* remained unchanged (data not shown and Supplementary Fig. 5a). To further confirm the importance of IL-22 in WNT inhibition, we administered recombinant IL-22.Fc to naive C57BL/6 mice and collected tissues after 24 h. Our data indicate reduced *Wnt6* expression in response to IL-22 stimulation (Supplementary Fig. 5b). Despite increased WNT levels, expression patterns of ISC-specific *Lgr5* and stemness markers (*Olfm4* and *Ascl2*) remained unchanged (Fig. 5c). Consistent with this observation, *Ki67* expression and the number of Ki67^+^ proliferative cells were comparable in both groups (Fig. 5d). In addition, Paneth cells via ISCs are important for promoting organoid growth in vitro.^22^ We cultured primary small intestinal organoids from naive *Il22Ra1*^*fl/fl*^*;Defa6-cre-* and cre+ mice and stimulated them with recombinant IL-22.Fc in the presence or absence of Wnt3a or NOTCH ligand DLL1. Consistent with previously published work,^12^ we show that IL-22 stimulates organoid growth. We specifically observed that organoids of control cre- mice but not in *Il22Ra1*^*fl/fl*^*;Defa6-cre*+ mice (Fig. 6a–c) grew in response to IL-22. The difference in organoid size in response to IL-22 stimulation started to appear on day 4 onwards (Fig. 6c). Based on our confocal microscopy results, Paneth cell numbers were comparable in both groups at days 2, 4, and 6 post culture (Fig. 6d). Interestingly, WNT3a or DLL1 alone or in combination with IL-22 does not influence organoid growth (Fig. 6a, b). Collectively, our data suggest that IL-22 acts specifically on Paneth cells to modulate the expression of selective WNTs as well as promote organoid growth in vitro.

### Paneth cell-intrinsic IL-22Ra1 signaling is required for microbiota (including SFB) colonization, small intestinal IL-17A and IL-22 responses, and anti-*Salmonella* immunity

**Fig. 7:**
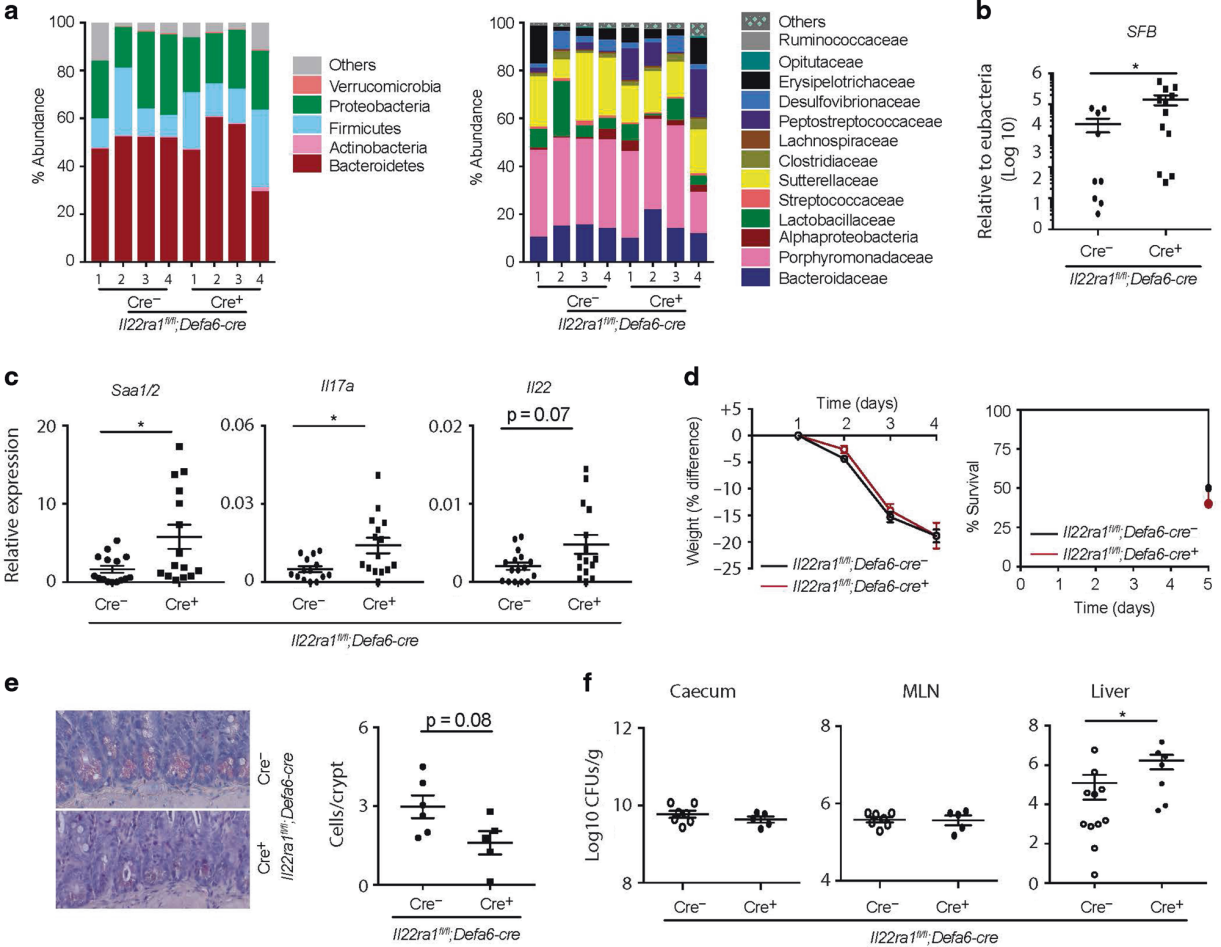
Paneth-specific IL-22Ra1 signaling prevents commensal dysbiosis (indicated by increased colonization by SFB) and is required for providing immunity against *Salmonella*. **a** Terminal ileum luminal contents of cohoused littermate *Il22Ra1*^*fl/fl*^*;Defa6-cre*+*/−* mice were analyzed for commensal diversity at the phyla (left panel) and selected family (right panel) levels by 16 S microbial sequencing. **b**, **c** RT-PCR analysis of SFB levels as well as *Saa1/2, Il17a* and *Il22* expression from ileal tissues of *Il22Ra1*^*fl/fl*^*;Defa6-cre*+*/−* mice. **d** Weight changes (left panel) and mortality curve (right panel) of *S*. Typhimurium infected *Il22Ra1*^*fl/fl*^*;Defa6-cre*+*/−* mice are shown**. e** Phloxine-tartrazine staining and quantification of Paneth cells in *Il22Ra1*^*fl/fl*^*;Defa6-cre*+*/−* mice on day 5 post *S*. Typhimurium infection. **f** *S*. Typhimurium burden in the caecum, MLN, and liver of *Il22Ra1*^*fl/fl*^*;Defa6-cre*+*/−* mice on day 5 post infection. Data are presented as mean ± SEM on relevant graphs. Figure 7b–f are generated from three independent experiments. Figure 7d is generated from 4–5 mice in each group. Figure 7e is a representative image of at least 5–6 mice in each group. Individual mouse data are shown in Fig. 7a. Data are presented as mean ± SEM on relevant graphs. *P ≤ 0.05 (Mann–Whitney test, two-tailed).

Given the reduced Paneth cell effector protein expression in the small intestine of naive *Il22Ra1*^*fl/fl*^*;Defa6-cre*+ mice, we next examined microbiota colonization levels in the terminal ileum lumen of these mice. High throughput 16S rRNA microbial community analysis of terminal ileum luminal contents of littermate co-housed mice was utilized to study the influence of Paneth cell-intrinsic IL-22Ra1 signaling on the overall microbial community. Analysis demonstrated that microbial communities remained largely unchanged in *Il22Ra1*^*fl/fl*^*;Defa6-cre*+ mice, suggesting a more subtle and specific control mechanism of Paneth cell-specific IL-22Ra1 signaling on the commensal microbial community (Fig. 7a left panel). The family level analysis revealed increased colonization of Alphaproteobacteria and Peptostreptococcaceae in the terminal ileum lumens of naive *Il22Ra1*^*fl/fl*^*;Defa6-cre*+ mice (Fig. 7a right panel). SFB colonization is critical for generating homeostatic IL-17A and IL-22 responses in the small intestine and its colonization is regulated by both IL-17A and IL-22.^3,29^ It remains unclear whether IL-22 signaling in Paneth cells is required for regulating colonization of SFB and homeostatic IL-17A and IL-22 responses. Our data show increased SFB colonization and elevated *Saa1/2* expression levels in the terminal ileum of naive *Il22Ra1*^*fl/fl*^*;Defa6-cre*+ mice (Fig. 7b, c). In line with these observations, we found increased *Il17a* and *Il22* expression in the small intestine of naive *Il22Ra1*^*fl/fl*^*;Defa6-cre* mice (Fig. 7c). Collectively, our data suggest that SFB and selective Proteobacteria spp. are regulated by IL-22Ra1 signaling in Paneth cells, but SFB-dependent IL-17A responses are regulated by non-Paneth cell epithelial lineages.

We next evaluated the role of Paneth cell-specific IL-22Ra1 signaling in a *S*. Typhimurium infection model where inflammation is evident in the colon and ileum. The mechanisms that regulate *S*. Typhimurium colonization are incompletely understood, especially since it is increasingly clear that IL-22 can act to promote^30^ or control (small intestine) its pathogenesis.^23–26^ It is possible that IL-22 acts via Paneth cells to mediate *S*. Typhimurium colonization. Also, IL-22-dependent protective and/or inflammatory responses may be mediated by Paneth cells. *Il22Ra1*^*fl/fl*^*;Defa6-cre*+ and their littermate cre- mice were fasted for 4 h and infected with 5 × 10^8^ CFU of *S*. Typhimurium. We found no difference in weight loss in *Il22Ra1*^*fl/fl*^*;Defa6-cre-* and cre+ mice (Fig. 7d left panel). However, mortality and pathological findings in the ileum were modestly elevated in *Il22Ra1*^*fl/fl*^*;Defa6-cre*+ mice (Fig. 7d right panel and Supplementary Fig. 5c). Paneth cell number was modestly reduced in the small intestine of *Il22Ra1*^*fl/fl*^*;Defa6-cre*+ mice after infection (Fig. 7e). While we found no difference in *S*. Typhimurium burden in the MLN and caecum of *Il22Ra1*^*fl/fl*^*;Defa6-cre*+ and their littermate cre- mice, we observed increased *S*. Typhimurium dissemination in the liver of *Il22Ra1*^*fl/fl*^*;Defa6-cre*+ mice on day 4 post infection (Fig. 7f). Consistent with our unchallenged mice data, *Mmp7* expression remained reduced in the terminal ileum of *S*. Typhimurium-infected *Il22Ra1*^*fl/fl*^*;Defa6-cre*+ mice; however, differences in *Lyz1* expression were no longer observed (Fig. 4d and Supplementary Fig. 5d). We found no difference in the expression of selected inflammatory cytokines (*Il1β* and *Tnfα*) of *S*. Typhimurium-infected *Il22Ra1*^*fl/fl*^*;Defa6-cre*+ mice (Supplementary Fig. 5d). It is possible that IL-22 signaling in non-Paneth cell types is required for protective responses. Indeed, global *Il22*^*−/−*^ mice have increased expression of inflammatory cytokines *Il1β* and *Tnfα* in the terminal ileum on day 4 post *S*. Typhimurium infection (Supplementary Fig. 5e). Upon infection, *Salmonella* selectively induces certain WNTs (WNT3a, WNT6 and WNT9a) to promote ISC survival.^31^ Next, we infected tamoxifen-administered *Il22Ra1*^*fl/fl*^*;Lgr5-EGFP-cre*^*ERT2+*^ and littermate cre- mice with *Salmonella*. We found *Il22Ra1*^*fl/fl*^*;Lgr5-EGFP-cre*^*ERT2+*^ were highly susceptible to infection with 100% mortality 5 days post infection as well as elevated, albeit not significant, bacterial dissemination to the liver (Supplementary Fig. 5f, g). Collectively, our data suggest IL-22Ra1 signaling in Paneth cells and ISCs may regulate systemic *S*. Typhimurium infection.

## Discussion

Paneth cells and their antimicrobial products (α-defensins, lysozyme, etc.) play a critical role in small intestinal host defense to injury or infection, and their dysregulation constitutes a pathogenic factor for Crohn’s Disease.^13–15^ Crohn’s patients have Paneth cell abnormalities and elevated levels of IL-22 in inflamed tissue.^14,16,17^ However, how IL-22 regulates Paneth cell function remains poorly understood. In this study, we present evidence indicating that IL-22Ra1 signaling in Paneth cells is required for small intestinal mucosal host defense and provides immunity against the bacterial pathogen, *S*. Typhimurium.

We observed that Paneth cell differentiation, development, and maturation positively correlate with IL-22 responses in the small intestine. Paneth cells and other epithelial cell lineages develop from Lgr5^+^ ISCs.^32^ Paneth cell differentiation starts postnatally on day 7; by day 42, they are fully differentiated and matured at the base of intestinal crypts, appropriately matched with microbiota establishment post weaning.^33^ A fully matured Paneth cell contains large secretory granules packed with antimicrobial peptides. Interestingly, IL-22 expression exactly follows the Paneth cell differentiation program in the small intestine suggesting IL-22 may regulate Paneth cell differentiation, maturation, or maintenance. Indeed, studies have shown that type 3 innate immune cell (ILC3)-derived IL-22 plays an important role in microbiota colonization immediately after weaning.^34^ This is evidenced by the phosphorylation of STAT3 (downstream of IL-22) in the entire crypt and villus epithelial cells and ILC3s. However, it was not clear whether IL-22Ra1 signaling in Paneth cells is required for microbiota colonization.

While we found a modest reduction in Paneth cell number in entire gut-epithelium-specific IL-22Ra1 knockout mice, Paneth cell-specific antimicrobial peptide expression was significantly reduced in *Il22Ra1*^*fl/fl*^*;Villin-cre*+ and germ-line *Il22*^*−/−*^ mice. Therefore, it is possible that Paneth cell differentiation and antimicrobial activity are regulated by distinct pathways. It is predicted that IL-22 regulates Paneth cell differentiation and maintenance which may account for the reduced *Lyz1* and *Mmp7* expression. Published literature suggests that the expression of key antimicrobial peptides is independent of transcriptional regulation rather than dependent on Paneth cell differentiation pathways.^27^ Consistent with a recent study,^35^ our data suggest that *Lyz1* and *Mmp7* expression are regulated at the transcriptional level. While our data suggest that Paneth cell number is not significantly affected by IL-22Ra1 signaling, IL-22 has been shown to promote Lgr5^+^ ISC-mediated epithelial cell proliferation and regeneration.^12,18^ Thus, it is possible that within a shorter duration of IL-22 stimulation (24 h), Lgr5^+^ ISCs are re-programmed to promote the differentiation of Paneth and other secretory cells. In addition, the majority of published work investigating the effects of IL-22 on intestinal ISC function was done in organoid-based assays, confounding ISC-related interpretation of data. Only a single study used ISC-specific IL-22Ra1 knockout mice.^18^ However, this study was not focused on ISC-mediated intestinal secretory lineage commitment or their functions in *Il22Ra1*^*fl/fl*^*;Lgr5-EGFP-cre*^*ERT2*^ mice.

Paneth cell-related results from *Il22Ra1*^*fl/fl*^*;Villin-cre*+ mice may be driven by IL-22Ra1 signaling in ISCs. However, we did not see a difference in Paneth cell number or expression patterns of selected antimicrobial genes in *Il22Ra1*^*fl/fl*^*;Lgr5-EGFP-cre*^*ERT2+*^ mice. A longer duration of tamoxifen may result in Paneth cell-related changes in *Il22Ra1*^*fl/fl*^*;Lgr5-EGFP-cre*^*ERT2*^ mice, but this approach has an inherent caveat whereby all ISC descendent cells including Paneth cells become IL-22Ra1 negative. Goblet, tuft, and enteroendocrine cell-related results in *Il22Ra1*^*fl/fl*^*;Lgr5-EGFP-cre*^*ERT2*^ mice suggest that IL-22 does not regulate secretory cell lineage commitment. A lineage-tracing experiment may be useful to determine the impact of IL-22 on ISCs to regulate Paneth cell differentiation. ATOH1 is required for intestinal secretory cell lineage commitment, and it may be possible that IL-22 acts on ATOH1^+^ transit-amplifying cells (TA) to mediate a role in Paneth cell differentiation. A recent study shows that IL-22 depletes ISCs but enhances TA cell proliferation and Paneth cell differentiation.^35^ It remains unclear whether effects mediated by secretory ATOH1^+^ progenitor TA cell-specific IL-22 are required for Paneth cell differentiation. Our *Il22Ra1*^*fl/fl*^*;Lgr5-EGFP-cre*^*ERT2*^ mice data coincide with this study indicating that IL-22 does not influence ISC function under homeostatic conditions. We also observed increased expression of *Olfm4* in tamoxifen-administered *Il22Ra1*^*fl/fl*^;*Lgr5-EGFP-cre*^*ERT2*^ mice, suggesting IL-22 reduces ISC stemness (data not shown). Interestingly, TA cells express low levels of *Lgr5*, and *Il22Ra1*^*fl/fl*^*;Lgr5-EGFP-cre*^*ERT2*^ mice display compromised IL-22Ra1 signaling in ATOH1^+^ TA cells. This raises the question regarding the role of TA cells in promoting Paneth cell differentiation.

One report showed that IL-22Ra1 is expressed on Lgr5^+^ stem cells rather than Paneth cells.^12^ It is possible that IL-22Ra1 expression is transient or dependent on local environments (ileum, jejunum, or duodenum). We used terminal ileum tissue from our conditional knockout mice to validate that expression of *Il22ra1* was reduced in the Paneth cells of our Paneth cell-specific IL-22Ra1 knockout mice (Fig. 4a and Supplementary Fig. 3). Our Paneth cell *Il22ra1* expression data are also supported by a recent study which shows that 72.7% of Paneth cells express *Il22ra1*.^19^

Our results obtained using Paneth cell-specific IL-22Ra1 knockout mice suggest that IL-22 has a specific and unique role in regulating Paneth cell maturation and antimicrobial effector functions. We did not observe as great of a difference in Paneth cell number in *Il22Ra1*^*fl/fl*^*;Defa6-cre*+ mice as in *Il22Ra1*^*fl/fl*^*;Villin-cre*+ mice. We and Juan-Min Zha et al. ruled out involvement of ISCs.^35^ It is possible that IL-22Ra1 signaling in stromal cells or secretory progenitors regulates Paneth cell differentiation. Stromal cell-derived growth factors may regulate TA cell proliferation. Indeed, PDGFRα^+^ stromal cells act via WNTs and R-spondin to regulate ISC function in the intestine.^36^ Investigation of the stromal cell-specific role of IL-22 in modulating ISC/TA cell niche and function will be useful for Paneth cell lineage commitment studies. Our study suggests that IL-22Ra1 signaling is required for Paneth cell maturation and effector function. More studies are required to dissect how IL-22 transcriptionally regulates Paneth cell antimicrobial effector genes. One possibility is that STAT3 binds to the promoter of *Lyz1*, *Mmp7*, and *Pancryptdin*. Indeed, MMP7 activity is regulated by STAT3 binding to its promoter in non-intestinal fibroblast cell lines.^37^

Interestingly, we also observed that IL-22 negatively regulates the gene expression of Paneth cell-specific selective WNTs. It has been previously shown that IL-22 inhibits WNT signaling in active ISCs.^35^ Here, we present similar results in Paneth cells. The exact mechanism of IL-22/WNT crosstalk is not very well understood. However, IL-22 can potentially increase the levels of WNT pathway inhibitor DKK1 which in turn negatively impacts the expression of WNT ligands. In addition, recent publications show tissue-dependent outcomes of the crosstalk between STAT3 and WNT signaling which suggests alternative modes of regulating the expression of WNT ligands.^38–40^ WNT proteins have been shown to regulate Paneth cell maturation and support the ISC maintenance ex vivo.^20–22^ However, in vivo functions of ISCs remain unaltered in the absence of Paneth cells.^41^ We also found no difference in ISC-specific gene expression, stemness factors, and Ki67^+^ proliferative cell number in *Il22Ra1*^*fl/fl*^*;Defa6-cre*+ mice despite elevated levels of WNTs. It is possible that stromal cell-derived WNTs may compensate for elevated Paneth cell-specific WNTs.

IL-22 inhibits the expression of WNTs in Paneth cells. R-spondin is a WNT agonist and is a necessary factor for organoid growth. Of note, in vitro organoid culture can be achieved without the addition of WNTs. Our data suggest that Paneth cell-dependent organoid growth is independent of WNT regulation by IL-22 (Fig. 6a, b). We show that IL-22 is required for optimum Paneth cell maturation and their antimicrobial activities. It is possible that the crypts of *Il22Ra1*^*fl/fl*^*;Defa6-cre*+ mice that we used for organoid assays possess immature and/or dysregulated Paneth cells. Therefore, this would not support an initial structure for proper IL-22-dependent organoid growth. It is also possible that an IL-22-mediated claudin flux occurs in Paneth cells (as reported in a recent study) and is required for organoid growth.^35^ Indeed, *Cldn1* is highly expressed in Paneth cells.^42^ Additional work is required to investigate the mechanisms of IL-22-mediated regulation of organoid growth.

IL-22 has been shown to regulate epithelial Reg3γ responses.^43^ Our data suggest that IL-22Ra1 signaling in Paneth cells either does not modulate *Reg3γ* expression or modulates its expression at very low levels. However, it is also possible that enhanced IL-22 responses in enterocytes of *Il22Ra1*^*fl/fl*^*;Defa6-cre*+ mice induce *Reg3γ* expression, thereby causing Paneth cell responses to be masked. Reg3γ protein levels in knockout mice may be required to further confirm our assumption.

We and others have shown that IL-22 is critical for SFB colonization and Th17 immune responses in the small intestine.^3,29^ We found increased SFB colonization in *Il22Ra1*^*fl/fl*^*;Villin-cre*+ mice, but expression of *Il17a* and *Il22* was unchanged. SAA1 has been shown to regulate SFB-dependent Th17 immune responses.^28^ Reduced *Saa1/2* levels in *Il22Ra1*^*fl/fl*^*;Villin-cre*+ mice may explain *Il17a* and *Il22* expression results. Reg3γ has been shown to regulate SFB colonization.^43^ However, SFB colonization was not altered despite a 50% reduction in *Il22Ra1* and *Reg3γ* expression in *Il22Ra1*^*fl/fl*^*;Lgr5-EGFP-cre*^*ERT2*^ mice. This suggests that IL-22Ra1 signaling in Paneth cells may be required for SFB colonization and Th17 immune responses in the ileum. Indeed, Paneth cell-specific IL-22Ra1 signaling is required for SFB and selected commensal microbiota colonization, but non-Paneth cell-specific IL-22 responses drive IL-17A and IL-22 responses in the small intestine. Additional work is required to reveal whether IL-22Ra1 signaling in enterocytes or non-Paneth secretory cells is required for IL-17A and IL-22 responses. We predict that IL-22-dependent regulation of *PanCryptidin* and *Mmp7* is responsible for SFB colonization. The importance of Paneth cells in controlling SFB has been evident by studies in HD5-transgenic mice and recombinant defcr25 administered mice.^13,29^

Our *S*. Typhimurium infection data show increased systemic bacteria dissemination in *Il22Ra1*^*fl/fl*^*;Defa6-cre*+ and tamoxifen-administered *Il22Ra1*^*fl/fl*^*;Lgr5-EGFP-cre*^*ERT2*^ mice. We believe dysregulation of Paneth cell-specific antimicrobial peptides, as well as impairment of the gut barrier, may have predisposed *Il22Ra1*^*fl/fl*^*;Defa6-cre*+ or *Il22Ra1*^*fl/fl*^*;Lgr5-EGFP-cre*^*ERT2+*^ mice to systemic *S*. Typhimurium dissemination. *Salmonella* manipulates WNT/β-catenin signaling pathways to increase ISC-dependent infectivity.^31^ In addition, a lineage trace study indicated that Paneth cells can acquire multipotency and regulate epithelial cell regeneration in the absence of ISCs.^44^ Additional work is required to investigate the mechanisms of systemic *S*. Typhimurium dissemination. Interestingly, we did not see any difference in the expression patterns of inflammatory cytokines in *Il22Ra1*^*fl/fl*^*;Defa6-cre*+ mice as we observed in global *Il22*^*−/−*^ mice in the terminal ileum of *Salmonella* infected mice. It is possible that IL-22 signaling in enterocytes, other secretory cells, or ISCs regulate inflammation. In addition, neutralizing or enhancing IL-22 function in *Il22Ra1*^*fl/fl*^*;Defa6-cre*+ mice may be useful to further dissect the cell lineage-specific functions of IL-22 in regulating *S*. Typhimurium infection.

Overall, our study highlights the importance of IL-22Ra1 signaling in Paneth cell lineages and its impact on the microbiome, immune responses, and pathogen colonization. These findings may have a great impact on our understanding of IL-22-dependent protective and/or inflammatory pathways. Likewise, results from this study may be beneficial in identifying a therapeutic target for multiple intestinal and extra-intestinal diseases.

## Materials and methods

### Mice

C57BL/6 (WT) and Villin-Cre (C57BL/6 background) were purchased from The Jackson Laboratory. Defa6-Cre mice were obtained from Dr. Richard Blumberg, Brigham and Women’s Hospital, Harvard. Generation and characterization of IL-22-Floxed (*Il22Ra1*^*fl/fl*^) mice was performed as described.^45^ *Rorc*^−/−^ and *Il22*^*−/−*^ (both C57BL/6 background) mice were received from Dr. Jay K. Kolls. *Il22Ra1*^*fl/fl*^ mice were bred with *Villin-cre*, *Lgr5-EGFP-cre*^*ERT2*^, or *Defa6-cre* mice to generate entire gut epithelium ISC-specific and Paneth cell-specific IL-22Ra1 knockout mice. *Lgr5-EGFP-cre*^*ERT2*^*;RosaLSLtdTomato* mice were obtained from Dr. Vincent Yang, Stony Brook University. We used 6–8 week aged mice (both genders) for all experiments unless indicated in the figure. All mice were housed in specific pathogen-free conditions at Stony Brook University, Stony Brook, NY. All animal studies were conducted with the approval of Stony Brook University Institutional Animal Care and Use Committee.

### RT-PCR

To isolate RNA from terminal ileum tissues, Trizol or Qiagen RNeasy kit was used following the manufacturer’s instructions. Biorad IScript kit was used to reverse transcribe RNA into cDNA. RT-PCR was performed using Biorad Sso advanced supermix. Applied Biosystem primer-probes for *Il17a* (Mm00439618_m1), *Il22* (Mm00444241_m1), *Hprt* (Mm00446968_m1), *Reg3γ* (Mm.PT.58.1275735), *Saa1/2* (Mm04208126), *Il22ra1* (Mm.PT.58.42129001), *Atoh1* (Mm00476035), and *Mmp7* (Mm00487724_m1), *Chga* (Mm0051431_m1), *Dclk1* (Mm00444950_m1), *Wnt6* (Mm00437351_m1), *Wnt9b* (Mm00457102_m1), *Ki67* (Mm_Mki67_1_SG), *Tgfb1* (Mm01178820_m1), *Egf* (Mm00438696_m1), and Integrated DNA Technologies^33^ primer-probes for *Muc2* (Mm.PT.58.53535475.g), *Tnfa* (Mm.PT.58.12575861), *Hes1* FP- 5′-TGCCTTTCTCATCCCCAACG-3′, *Hes1* RP – 5′-AGGTGACACTGCGTTAGGAC-3′, *Ascl2* FP 5′-CTACTCGTCGGAGGAAAG-3′, *Ascl2* RP 5′-ACTAGACAGCATGGGTAAG-3′, *Lyz1* (Mm.PT.58.7374112) were used. RT-PCR analysis for mammalian genes was calculated relative to *Hprt*. Quantitate relative abundance of SFB and total bacteria (Eubacteria) were determined using group-specific rDNA primers SFB736F—GACGCTGAGGCATGAGAGCAT, SFB844R—GACGGCACGGATTGTTATTCA, Eubact Uni340FP—ACTCCTACGGGAGGCAGCAGT, Eubact Uni514RP—ATTACCGCGGCTGCTGGC as described before.^13,46^ RT-PCR analysis for bacterial genes was analyzed relative to Eubacteria.

### Phloxine-tartrazine staining

Five micrometer thick paraffin-embedded tissue sections were deparaffinized and rehydrated using Xylene and a descending ethanol gradient (100%, 95%, 70%, pure dH_2_O). Mayer’s Haematoxylin solution was used to stain nuclei to a medium density (~1 min). Slides were thoroughly rinsed in running tap water for 5 min and stained with phloxine solution for 20 min. Slides were rinsed with tap water, blotted dry, and rinsed with tartrazine solution to remove remaining water. Slides were kept in tartrazine solution and visualized under a microscope until granules were red and all other tissue was yellow. Slides were dehydrated in 70, 95, and 100% ethanol and then placed in two washes of 100% xylene before being mounted. For analysis of average Paneth cell number per crypt, at least 10 crypts were counted per mouse. Images were acquired using a Zeiss Observer D1 Inverted Phase Contrast Fluorescence Microscope, Axiocam 105 color camera, and Zeiss Zen pro microscope software (Stony Brook University).

### Alcian blue staining

Five micrometer thick paraffin-embedded tissue sections were deparaffinized and rehydrated using Xylene and a descending ethanol gradient (100%, 95%, 70%, pure dH_2_O). Three percent acetic acid was applied to the slides for 3 min. Alcian blue solution (pH 2.5) was added to slides for 30 min to stain the goblet cells. Three percent acetic acid was applied to the slides for ~30 s to remove excess Alcian blue staining. Slides were rinsed in running tap water for 5 min followed by two changes of distilled water. Nuclear Fast Red solution was applied for 5 min to stain nuclei. Slides were rinsed with running tap water for 2 min, transferred to two changes of distilled water, and then dehydrated with an ascending ethanol gradient (70, 95, and 100%). Slides were mounted. Images were acquired using a Zeiss Observer D1 Inverted Phase Contrast Fluorescence Microscope, Axiocam 105 color camera, and Zeiss Zen pro microscope software (Stony Brook University).

### Tamoxifen treatment

Tamoxifen (1 mg/mouse) or corn oil was administered intraperitoneally for 5 consecutive days. On day 9 post last tamoxifen injection, *Il22Ra1*^*fl/fl*^*;Lgr5-EGFP-cre*^*ERT2*^ mice tissue was harvested for RT-PCR. On day 5 (last tamoxifen injection), *Il22Ra1*^*fl/fl*^*;Lgr5-EGFP-cre*^*ERT2+/−*^ mice were infected with *Salmonella*. On day 6 (1 day after last tamoxifen injection), *Il22Ra1*^*fl/fl*^*;Lgr5-EGFP-cre*^*ERT2+*^ mice tissues were used for IL-22Ra1 staining by flow cytometry. Tamoxifen (1 mg/mouse) was administered intraperitoneally for 5 consecutive to *Lgr5-EGFP-cre*^*ERT2*^*;RosaLSLtdTomato* mice.

### IL-22 treatment

Recombinant human IL-22.Fc (generously provided by Evive Biotech. China) was administered intraperitoneally (80 μg/mouse) in C57BL/6 mice or *Lgr5-EGFP-cre*^*ERT2*^*;RosaLSLtdTomato* mice. A dose response curve was generated to determine the optimal dose of IL-22 in inducing Reg3γ (data not shown). Mouse small intestine-derived organoids were continuously (starting on day 1 till day 8) treated with IL-22.Fc (4 ng/ml). Further details regarding organoid culture are discussed in a subsequent section.

### *S.* Typhimurium infection

*Il22Ra1*^*fl/fl*^*;Defa6-cre*+, tamoxifen-administered *Il22Ra1*^*fl/fl*^*;Lgr5-EGFP-cre*^*ERT2*^ and their littermate cre- mice were fasted for 4 h then gavaged with streptomycin (0.1 ml of a 200 mg/ml solution in sterile water administered orally) 24 h prior to oral gavage with 5 × 10^8^ CFU *Salmonella enterica* serovar Typhimurium strain IR715. After injection, mice were provided with food and water *ad libitum*. Mice were monitored for weight loss and mortality, and on day 4 or 5 mice were euthanized. Liver, caecum, and mesenteric lymph nodes were removed, homogenized in LB media, and plated on LB agar plates supplemented with nalidixic acid to evaluate systemic bacterial dissemination.

### 16S rRNA microbial community analysis

Terminal ileum luminal contents DNA from littermate cohoused *Il22Ra1*^*fl/fl*^*;Defa6-cre*+ mice and cre^−^ mice (6 weeks age) were isolated using QiAMP stool DNA extraction kit. Microbial community analysis utilized PCR amplification of the V4 region of 16S rRNA followed by sequencing on an Illumina MiSeq was performed and analyzed by MR DNA (Shallowater, Tx, USA). Sequences were analyzed using QIIME 1.8 as described.^47^ Differentially abundant OTUs were determined utilizing the group significance script within QIIME.

### Organoid culture

Roughly 10 cm of mouse small intestinal tissue (starting from the terminal ileum) was flushed with ice-cold 1× PBS, sectioned, and rinsed an additional four times with cold 1× PBS. The tissues were then cut into 0.5 cm pieces and transferred into 7.5 mM EDTA in 1× PBS. Tissues were incubated for 30 min on an orbital shaker (60 rpm) at 4 °C. After incubation, the tissues were washed to remove excess EDTA and were placed into cold 1× PBS. Tissues were gently shaken for 1 min and the supernatant was filtered through a 70 µm filter (BD biosciences) into a new conical tube. This was performed two additional times to achieve a total of three fractions. Each fraction was spot-checked and any fraction with a high amount of debris or low crypt yield was discarded. Filtered crypts were pelleted via centrifugation (2000 rpm for 5 min) and re-suspend in Matrigel hESC-qualified Matrix (Corning) at a concentration of 80 crypts per 20 µL Matrigel. Twenty microliters of this suspension were dispensed per well in 24-well plates. After polymerization of Matrigel for 30 min at 37 °C and 5% CO_2_, the solid matrix was overlaid with 500 µL medium containing growth factor R-Spondin (100 ng/ml) and Noggin (50 ng/ml) in advanced Dulbecco’s modified Eagle medium (DMEM)/F12, supplemented with 1× penicillin/streptomycin, 1× GlutaMAX, 1× N2, 1× B27 (all form Invitrogen).

The following growth factors were added to the medium media prior to plating: human EGF 40 ng/mL (Peprotech), 1µM N-acetylcysteine (Sigma), 20 µM Y-27632 (Tocris) and 5.6 µM CHIR 99021 (Tocris). Media was changed every 2 days.

For C57BL/6 organoids, a single-cell suspension was prepared on day 7 and was sorted. Defa6-Cre organoids were stimulated with 4 ng/mL IL-22.Fc, 100 ng/mL recombinant mouse Wnt3a (R&D) or 1 μg/mL recombinant mouse DLL1.Fc (R&D) wherever indicated. Images were taken 2, 4, and 6 days after plating using an Olympus CKX41 microscope and GRYPHAX imaging camera and software.

### RNA sequencing (RNA-seq)

Organoid: Primary organoids from C57BL/6 mice were cultured as described above. Cells were dissociated using TrypLE Express and single cells were directly sorted into a 96-well plate. Total RNA was used as starting material for deep sequencing.

#### Tissue

RNA from the terminal ileum of 6-week-old *Il22Ra1*^*fl/fl*^*;Defa6-cre*+ and their littermate cre- mice were isolated using TRIZOL extraction method as per manufacture instruction (Life Technologies).

### Single-cell RNA sequencing (scRNA-seq)

Primary organoids from C57BL/6 mice were generated as described above. Four replicates of organoids were pooled together from 1 mouse for scRNA-seq. Cells were dissociated using TrypLE Express, washed with 1× PBS with 0.4% BSA and stored on ice until proceeding to single-cell capture for RNA sequencing. Single cells were processed through the Chromium Single Cell Platform using the Chromium Single Cell 3′ Library, Gel Bead and Chip Kits (10X Genomics, Pleasanton, CA), following the manufacturer’s protocol. In brief, an input of 10,000 cells was added to each channel of a chip with a recovery rate of 3000 cells. The cells were then partitioned into Gel Beads in Emulsion in the Chromium instrument, where cell lysis and barcoded reverse transcription of RNA occurred, followed by amplification, tagmentation and 5′ adaptor attachment. Libraries were sequenced on an Illumina NextSeq 500. Alignment to the mm10 mouse genome and unique molecular identifier (UMI) collapsing was performing using the Cellranger toolkit (version 1.3.1, 10X Genomics). For each cell, we quantified the number of genes for which at least one UMI was mapped and then excluded all cells with fewer than 1000 detected genes.

### Total RNA sequencing

#### Illumina TruSeq stranded total RNA library prep with ribo-zero gold ribosomal depletion

Prior to Illumina TruSeq Total RNA library construction, Total RNA was quantitated using the Qubit RNA BR assay kit (Thermo Fisher Scientific: Guide MAN0001987 MP10210, Kit #Q10210). RNA quality was determined on an Agilent 4150 Tapestation (G2992AA) using Agilent RNA ScreenTape Analysis (Agilent: Publication Part Number: G2991-90021, Kit #5067-5576). 2.5 μg Total RNA was treated with 1MBU Baseline-Zero DNase (Epicentre Cat. No DB0711K) following the General Baseline –Zero DNase protocol (www.epicentre.com, Lit # 263). RNA was purified and concentrated using 1.8 × vol Agencourt RNAClean XP Beads (Beckman Coulter A63987) following the standard protocol (Protocol 001298v001). Concentrations of samples were again measured with Qubit BR RNA assay kit for input into the TruSeq Stranded Total RNA library protocol.

Illumina compatible Total RNA cDNA libraries with RiboZero Gold depletion were generated following the TruSeq Stranded Total RNA Reference Guide (Illumina Document #1000000040499v00, TruSeq Stranded Total RNA Gold Illumina Kit #20020598). Final cDNA libraries were quantitated using Qubit dsDNA BR assay kit (Thermo Fisher Scientific: Guide MAN0002325 MP 32850, Kit #Q32850). The quality of the libraries was determined by running each on an Agilent 4150 Tapestation using the DNA 1000 Screentape kit (Agilent: Publication Part Number: G2991-90031, Kit # 5067-5583). Region analysis was performed on the electropherogram of each library using Agilent 4150 Tapestation Analysis Software (Version 3.1) with a range of 200–600 bp to determine average size of each library. Average size and concentration were then used to calculate the molarity of each library. All libraries were pooled and denatured following the standard normalization method in the Illumina Denature and Dilute Libraries Guide for the NextSeq System (Illumina Part #15048776). Finally, denatured libraries were loaded onto an Illumina NextSeq 550 v2.5 Mid 150 output reagent cartridge (Illumina # 20024904) at a final concentration of 1.5pM in HT1 buffer. Illumina Denatured PhiX control library v3 (Illumina #FC-110-3001) was also included at 1% concentration. Paired end 75 bp single index sequencing was performed yielding ~7.5 M PE reads per sample.

Sequencing analysis was done using RNA-seq for Eukaryotes Analysis v3 by Banana Slug Genomics Center at University of California Santa Cruz. Raw sequencing reads (paired-end reads) that were produced by Illumina sequencer were quality checked for potential sequencing issues and contaminants using FastQC.^1^ Adapter sequences and primers were trimmed from the sequencing reads using Trimmomatic,^2^ then followed by removing polyA tail, polyN, and read portions with quality score below 28 using PRINSEQ. Reads with a remaining length of fewer than 20 bp after trimming were discarded. A second round of quality check with FastQC was made to compare read quality before and after trimming. Trimmed reads were mapped to the reference genome (GRCm38/mm10) using (TopHat2) with NCBI RefSeq annotated genes as transcriptome index data. Read alignment coverage and summary statistics for visualization were computed using SAMtools, BEDtools, and UCSC Genome Browser utilities. Cufflinks 2.2.0 workflow and read-counting methodology with DESeq and edgeR were adopted for abundance estimation and differential expression analysis. In brief, using Cufflinks workflow, the sequencing reads aligned to RefSeq annotated genes were quantified using Cuffquant. Cuffnorm was then used to normalize the gene expression levels across the studied samples with FPKM computed for sample correlation assessment. Differential expression analysis between samples was performed using Cuffidff with the computed results from Cuffquant. Using read-counting methodology, HTSeq was adopted to compute raw read counts for annotated RefSeq genes. Raw read counts were normalized across all samples and then used for differential expression analysis using edgeR.

### Immunofluorescence

For staining, terminal ileum tissue was fixed in 10% formalin and embedded in paraffin blocks. Paraffin-embedded tissue sections (5 μm) were deparaffinized using Xylene and rehydrated using a descending ethanol gradient. Antigen retrieval was conducted by heating slides in a microwave. Tissue sections were blocked in 5% bovine serum albumin in 1× PBS for 1 h at 37 °C. Tissue sections were incubated at 4 °C overnight in primary antibodies against LYZ1-FITC (Dako, 1:200), MMP7 (Cell Signaling Technology, 1:200). Slides were washed three times for 5 min, and sections were incubated with anti-rabbit IgG Fab2 Alexa Fluor® 647 (Cell Signaling Technology, 1:300). Slides were mounted using a DAPI hard stain (Vector® Laboratories, H-1500) to visualize cell nuclei. Images were acquired using a Zeiss 510 Meta NLO confocal microscope (Stony Brook University).

For lineage-tracing experiments, Rabbit anti-RFP (Rockland, 1:300) was used as a primary antibody, Mouse anti-rabbit (Jackson Laboratories, 1:300) was used as a secondary antibody and Donkey anti-mouse-Cy5 (Jackson Laboratories, 1:300) was used as a tertiary antibody. The primary antibody was applied overnight at 4 °C, and the secondary and tertiary antibodies were applied for 30 min at 37 °C. Slides were washed three times for 5 min after each antibody was applied.

### Transmission electron microscopy

*Il22Ra1*^*fl/fl*^*;Defa6-cre*+ and cre- mice terminal ileum samples were used for transmission electron microscopy and were processed using standard techniques. In brief, samples were fixed overnight in Karnovsky’s Fixative (Electron Microscopy Sciences). Samples were then placed in 1% osmium tetroxide in 0.1 M PBS pH 7.4, dehydrated in a graded series of ethyl alcohol and embedded in EMbed 812 resin. Ultrathin sections of 80 nm were cut with a Leica EM UC7 ultramicrotome and placed on formvar coated slot copper grids. Sections were then counter-stained with uranyl acetate and lead citrate and viewed with a FEI Tecnai12 BioTwinG^2^ transmission electron microscope. Digital images were acquired with an AMT XR-60 CCD Digital Camera system.

### Flow cytometry

The small intestines from *Il22Ra1*^*fl/fl*^*;Villin-Cre*,tamoxifen-treated *Il22Ra1*^*fl/fl*^*;Lgr5-EGFP-cre*^*ERT2+*^,*Il22Ra1*^*fl/fl*^*;Defa6-Cre* or *Lgr5-EGFP-cre*^*ERT2*^*;RosaLSLtdTomato* mice were harvested. The ileum or entire small intestine were separated from the mesentery and Peyer’s patches were carefully excised. Tissues were flushed with ice-cold 1× PBS and opened longitudinally. Tissues were washed four additional times with ice-cold PBS and were cut into ~0.5 cm pieces Epithelial cells were separated from the lamina propria by incubating the tissue pieces in 7.5 mM EDTA in 1× PBS on an orbital shaker (60 rpm) for 30 min at 4 °C. Crypts were released by shaking tissue pieces in 1× PBS and repeating this step three. Crypts were filtered using a 70-μm cell strainer and pelleted by centrifugation at 2000 rpm for 5 min at 4 °C. The crypts were incubated for 15 min at 4 °C in fetal bovine serum (10%) containing DMEM/F12 medium. Cells were dissociated using TrypLE Express (Invitrogen) supplemented with 10 μM Rock inhibitor (Y-27632) and 2.5 μg/ml DNAse 1 (Sigma-Aldrich) for 5 min at 37 °C. Cells were filtered using a 70-μm cell strainer to remove clumps and mucus. The cells were washed twice with PBS and pelleted by centrifugation at 4 °C at 2000 rpm for 3 min. Cell suspensions were stained with IL-22Ra1 (R&D), UEA-1 (Vector), CD24 (eBioscience), and EpCAM (eBioscience) antibodies for acquisition at BD-LSR-Fortessa. Cell pellets from tamoxifen-treated *Il22Ra1*^*fl/fl*^*;Lgr5-EGFP-cre*^*ERT2+*^ mice were re-suspended in 600ul PBS for sorting.

## Supplementary information

Supplementary Figures

## Data Availability

All RNA-seq data have been deposited in the GEO database (GSE159423) and all 16s files have been deposited in the SRA database (SAMN16424612).
