# Supplementary material for: IL-22 receptor signaling in Paneth cells is critical for their maturation, microbiota colonization, Th17-related immune responses, and anti-Salmonella immunity
Source: Mucosal Immunol. 2020 Oct 15;14(2):389–401. doi: 10.1038/s41385-020-00348-5 (PMC7946635; doi:10.1038/s41385-020-00348-5)
Supplement: Supplementary file 1 — Supplementary Figures [file 41385_2020_348_MOESM1_ESM.pdf]

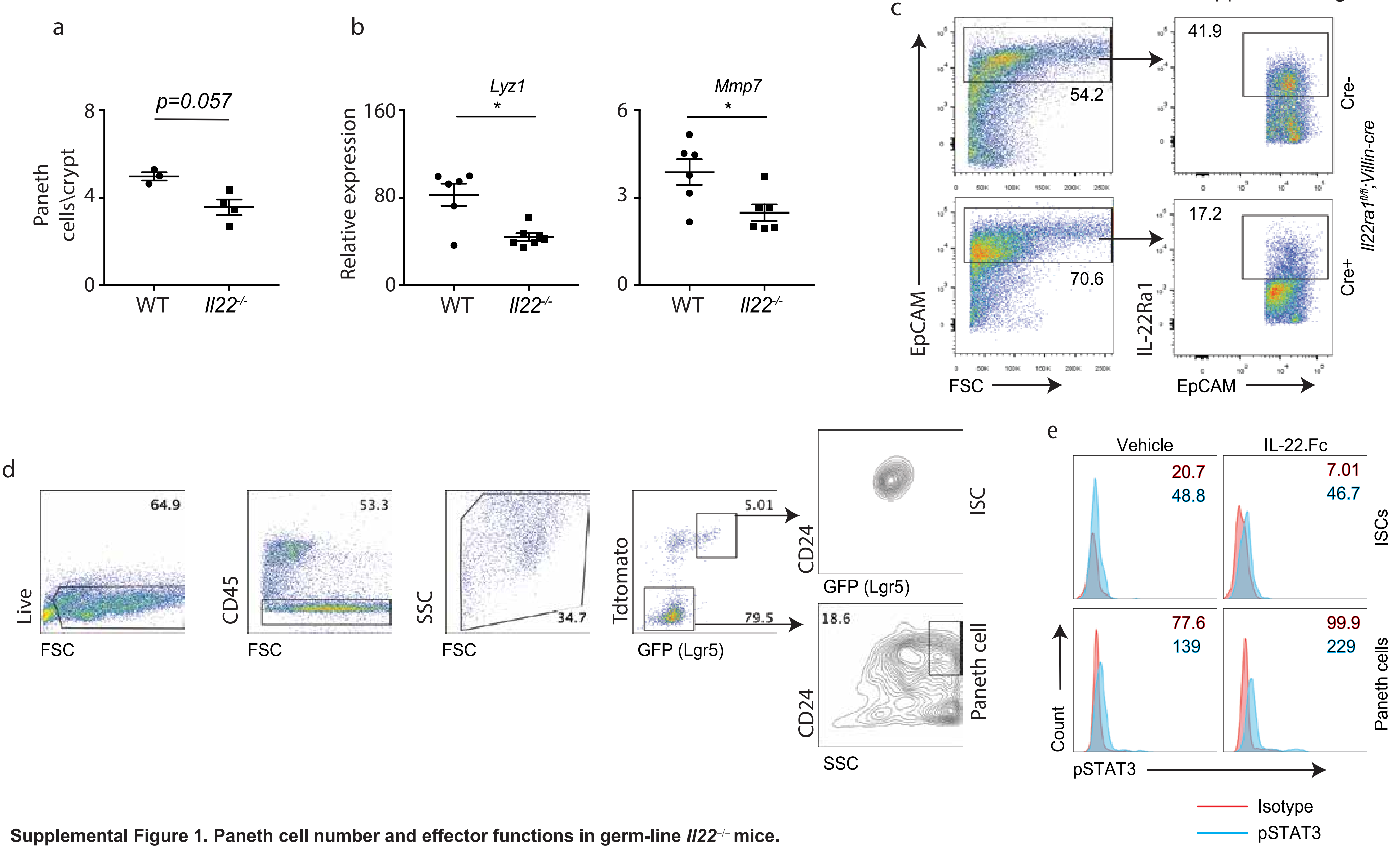

Supplemental Figure 1. Paneth cell number and effector functions in germ-line *Il22*$^{-/-}$ mice.
A) Phloxine-tartrazine staining and quantification of Paneth cells in naïve *Il22*$^{-/-}$ mice.
B) Terminal ileum RT-PCR analysis displaying *Lyz1* and *Mmp7* expression in cohoused naïve C57BL/6 and *Il22*$^{-/-}$ mice at 6 weeks age.
C) Flow cytometry data showing IL-22Ra1 production in the terminal ileum of *Il22Ra1*$^{fl/fl}$*;Villin-cre+/-* mice.
D) Flow cytometry data showing the gating strategy of ISCs and Paneth cells in *Lgr5-EGFP-cre*^{ERT2}*;RosaLSLtd*^{Tomato} mice.
E) Flow cytometry data showing pSTAT3 staining of ISCs (GFP^{high}) and Paneth cells (GFP^{-}, CD45^{-}, Tdtomato-CD24^{+}) from the small intestine of tamoxifen administered *Lgr5-EGFP-cre*^{ERT2}*;RosaLSLtd*^{Tomato} mice in response to vehicle (PBS) or IL-22.Fc stimulation (80 µg/mouse).
Supplemental figure 1C is a representative image from 2 mice per group. Supplemental figures 1D and E are generated from 2-2 mice per group.
Data are presented as mean ± SEM on relevant graphs. *P ≤ 0.05 (Mann-Whitney test, Two-tailed)

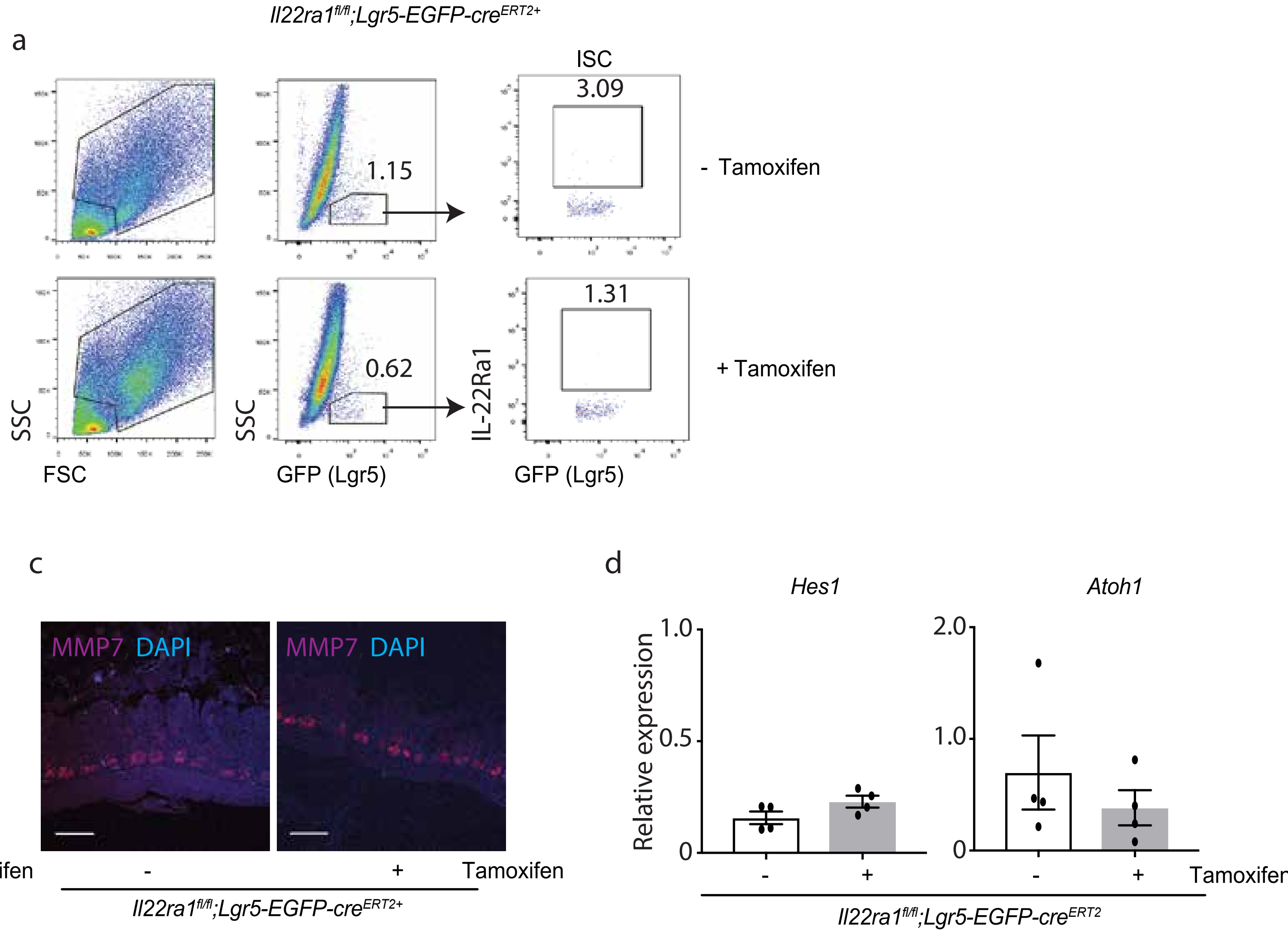

Supplemental Figure 2. IL-22Ra1 signaling in intestinal stem cells is dispensable for Paneth cell functions and epithelial cell lineage commitment.
A) Flow cytometry data showing IL-22Ra1 production on ISCs and EpCAM+ cells in the terminal ileum of *Il22Ra1$^{fl/fl}$;Lgr5-EGFP-cre^{ERT2+}* mice.
B and C) Immunofluorescence analysis of Lyz1 (B) and MMP7 (C) from ileal tissues of corn oil or tamoxifen administered *Il22Ra1$^{fl/fl}$;Lgr5-EGFP-cre^{ERT2+}* mice.
D) RT-PCR analysis of *Hes1* and *Atoh1* expression from ileal tissues of corn oil or tamoxifen administered *Il22Ra1$^{fl/fl}$;Lgr5-EGFP-cre^{ERT2+}* mice.
Supplemental figure 2A is generated from 2 mice in each group. Supplemental figures 2B and 2C are representative images of at least 3-4 mice in each group.
Supplemental figure 2D is generated from two independent experiments. Data are presented as mean ± SEM on relevant graphs.
Scale bars in relevant figures equal 100 μm.

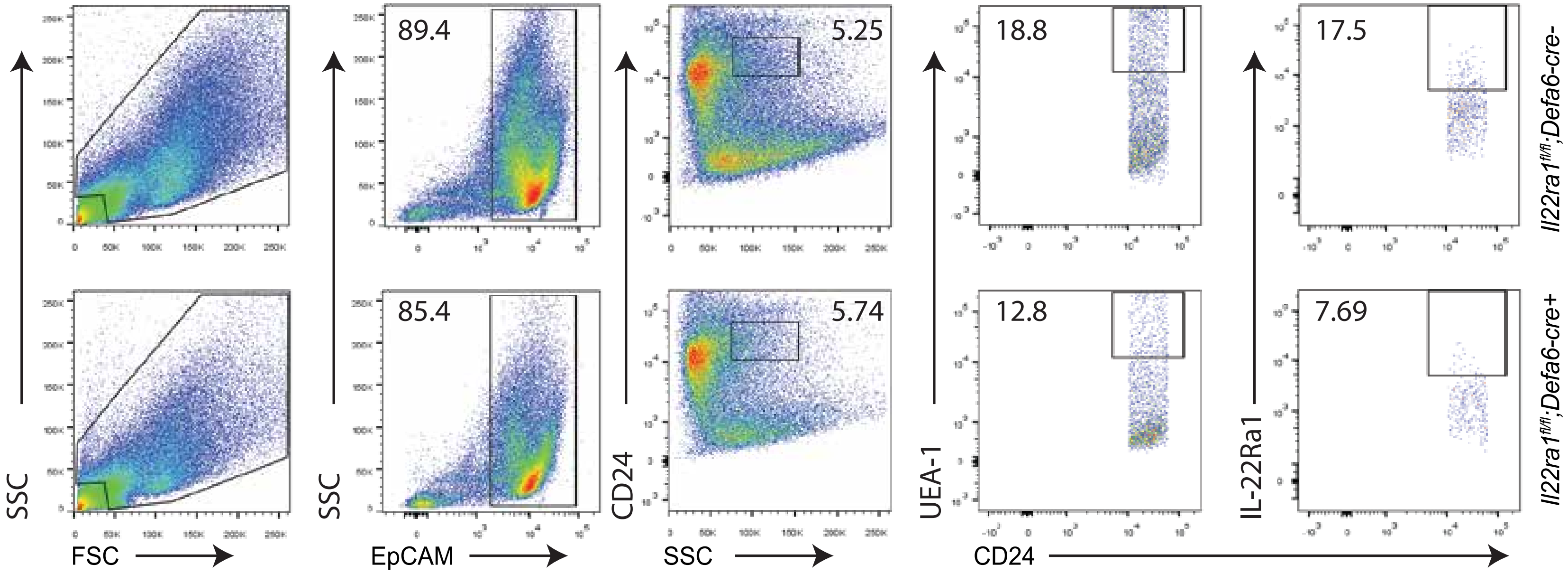

Supplemental Figure 3. IL-22Ra1 expression in EpCAM⁺UEA-1⁺CD24⁺ cells in *Il22Ra1$^{fl/fl}$;Defa6-cre+/-* mice.
Flow cytometry data showing IL-22Ra1 expression on EpCAM⁺UEA-1⁺CD24⁺ cells in the small intestine of *Il22Ra1$^{fl/fl}$;Defa6-cre+/-* mice. Flow plots are representative images of two mice in each group.

Supplemental figure 4

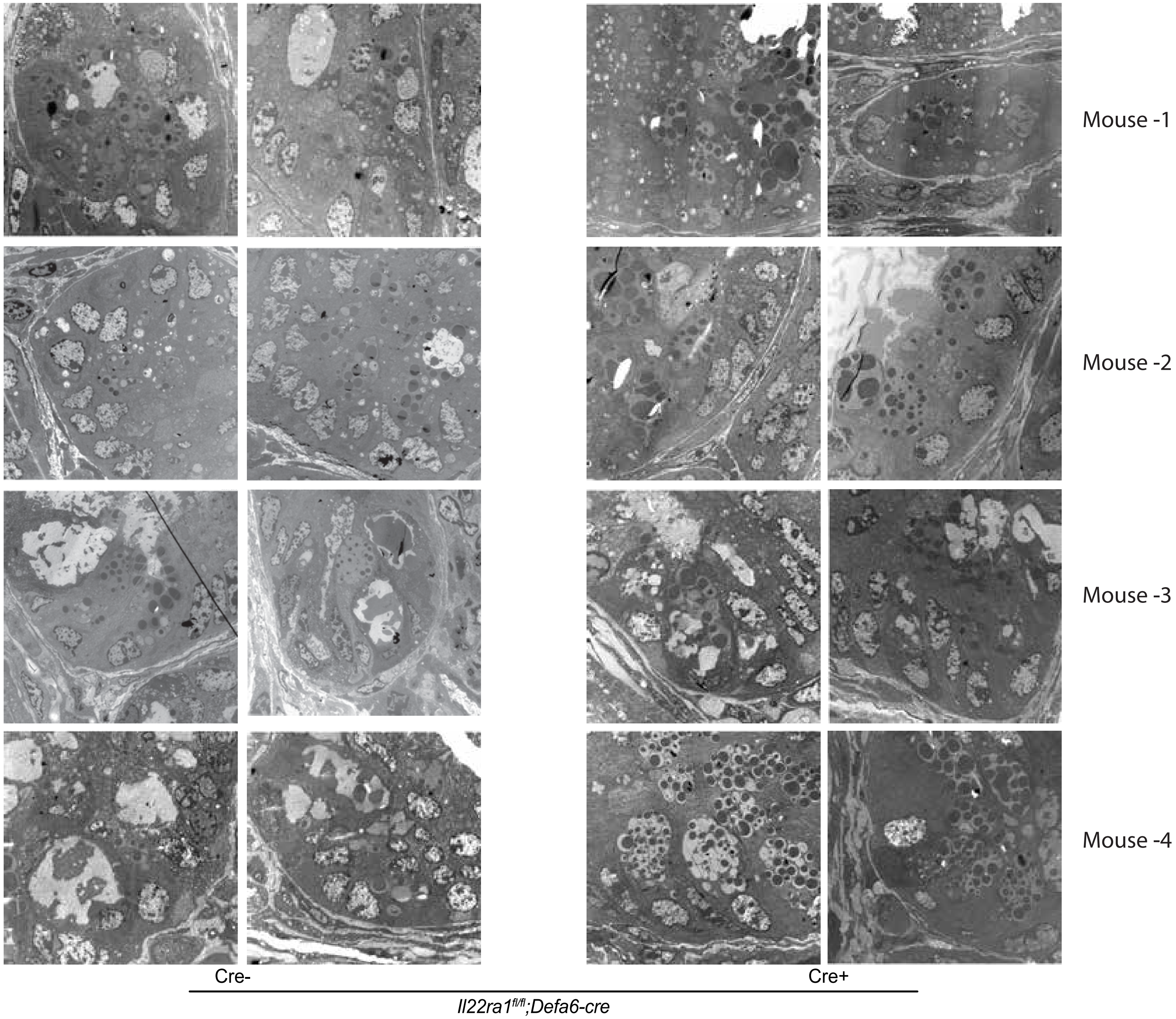

Supplemental Figure 4. Dysregulated granule size in *Il22Ra1$^{fl/fl}$;Defa6-cre+* mice.
TEM images of Paneth cells demonstrating secretory vesicles (2900x) in the terminal ileum of *Il22Ra1$^{fl/fl}$;Defa6-cre*+/- mice.
Data shown (2 images) is generated from 4 mice in each group.

Supplemental figure 5

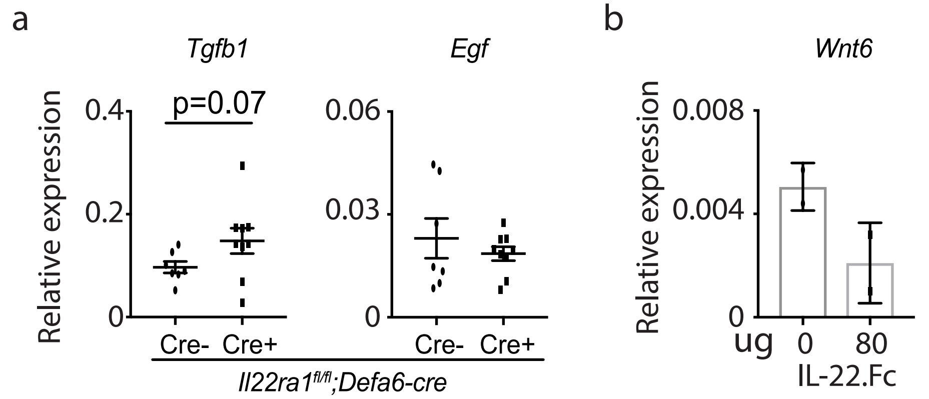

c

Genotype	Mouse	Extent	Seveity		Other notes
			Acute (Neutrophilic)	Chronic (Lymphoid)	
Il22ra1fl/fl;Defa6-cre-	*1*	*Negative*	*Negative*	*Negative*	
	2	*Negative*	*Negative*	*Negative*	
	3	*Weakly positive*	*Mild*	*Negative*	
	4	*Weakly positive*	*Severe*	*Negative*	
Il22ra1fl/fl;Defa6-cre+	*1*	*Negative*	*Negative*	*Negative*	
	2	*Negative*	*Negative*	*Negative*	*Edema in villi tips*
	3	*Negative*	*Negative*	*Negative*	*Inflammatory debris in lumen*
	4	*Weakly positive*	*Negative*	*Severe (granulomatous inflammation)*	*Inflammatory debris in lumen*
	5	*Weakly positive*	*Negative*	*Severe (granulomatous inflammation)*	*Inflammatory debris in lumen*

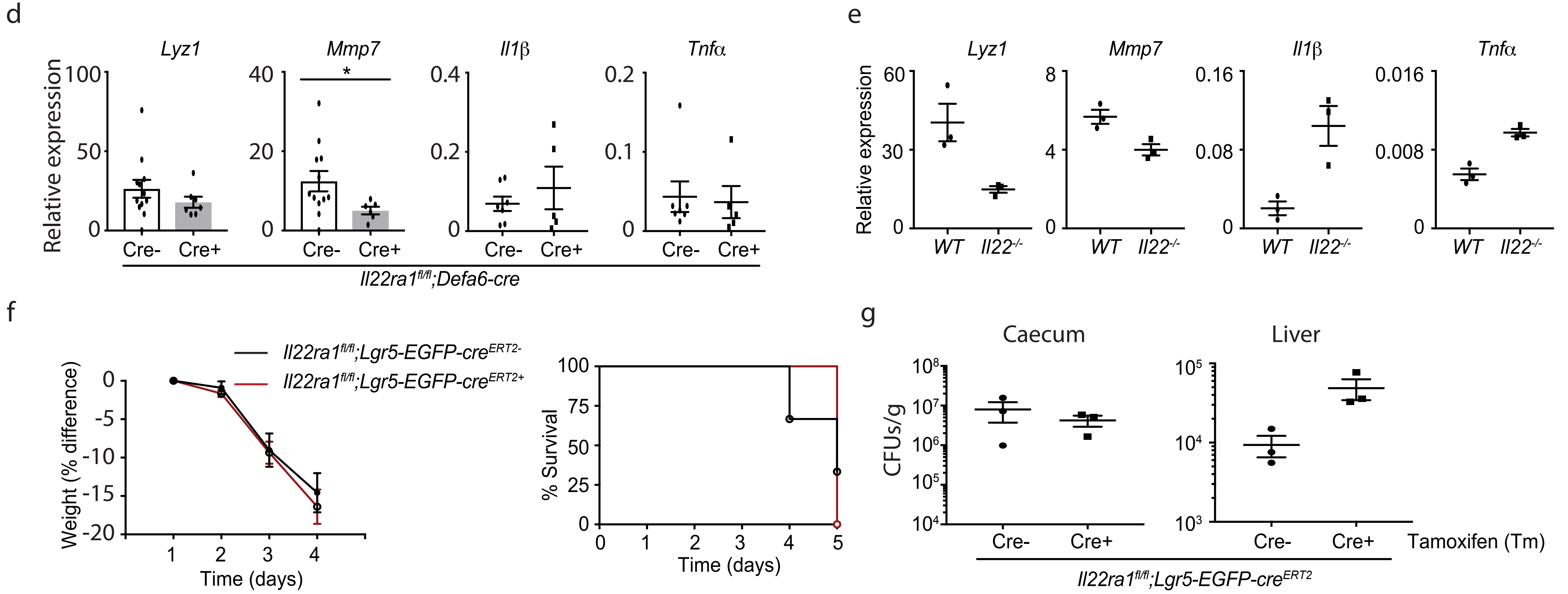

Supplemental Figure 5. *Il22*$^{-/-}$ and *Il22Ra1*$^{fl/fl}$*;Lgr5-EGFP-cre*$^{ERT2+}$ mice are more susceptible to S. *Typhimurium.*

A) RT-PCR analysis of *Tgfb1* and *Egf* expression from terminal ileal tissues of *Il22Ra1*$^{fl/fl}$*;Defa6-cre+/-* mice.

B) *Wnt6* expression in the ileal tissues of C57BL/6 mice 24 hours post IL-22.Fc administration.

C) Pathological evaluation of day 4 S. *Typhimurium* infected *Il22Ra1*$^{fl/fl}$*;Defa6-cre+/-* mice terminal ileal tissues.

D) RT-PCR analysis of *Lyz1, Mmp7, Il1*β and *Tnf*α expression from terminal ileal tissues of *Il22Ra1*$^{fl/fl}$*;Defa6-cre*$^{+/-}$ mice on day 5 post S. *Typhimurium* infection.

E) RT-PCR analysis of *Lyz1, Mmp7, Il1*β and *Tnf*α expression from terminal ileal tissues of WT and *Il22*$^{-/-}$ mice on day 5 post S. *Typhimurium* infection.

F) Weight loss (left panel) and mortality curve (right panel) of tamoxifen administered S. *Typhimurium*-infected *Il22Ra1*$^{fl/fl}$*;Lgr5-EGFP-cre*$^{ERT2+/-}$ mice.

G) S. Typhimurium burden in the caecum and liver of tamoxifen administered and infected *Il22Ra1*$^{fl/fl}$*;Lgr5-EGFP-cre*$^{ERT2+/-}$ mice on days 4 post infection.

Supplemental figures 5A, 5D and 5F are generated from two independent experiments. Data are presented as mean ± SEM on all graphs except Supplemental Figure 5B (mean ± SD). *P ≤ 0.05 (Mann-Whitney test, Two-tailed).
